# Investigation on Metabolites in Structure and Biosynthesis from the Deep-Sea Sediment-Derived Actinomycete *Janibacter* sp. SCSIO 52865

**DOI:** 10.3390/molecules28052133

**Published:** 2023-02-24

**Authors:** Wenping Ding, Yanqun Li, Xinpeng Tian, Zhihui Xiao, Ru Li, Si Zhang, Hao Yin

**Affiliations:** 1 CAS Key Laboratory of Tropical Marine Bio-Resources and Ecology, South China Sea Institute of Oceanology, Chinese Academy of Sciences, Guangzhou 510301, China; 2 Southern Marine Science and Engineering Guangdong Laboratory (Guangzhou), Guangzhou 511458, China; 3 Sanya Institute of Ocean Eco-Environmental Engineering, Sanya 572000, China

**Keywords:** *Janibacter* sp. SCSIO 52865, metabolites, structural elucidation, bioinformatic analysis, cyclodipeptides

## Abstract

For exploring structurally diverse metabolites and uniquely metabolic mechanisms, we systematically investigated the chemical constituents and putative biosynthesis of *Janibacter* sp. SCSIO 52865 derived from the deep-sea sediment based on the OSMAC strategy, molecular networking tool, in combination with bioinformatic analysis. As a result, one new diketopiperazine (**1**), along with seven known cyclodipeptides (**2**–**8**), *trans*-cinnamic acid (**9**), *N*-phenethylacetamide (**10**) and five fatty acids (**11**–**15**), was isolated from the ethyl acetate extract of SCSIO 52865. Their structures were elucidated by a combination of comprehensive spectroscopic analyses, Marfey’s method and GC-MS analysis. Furthermore, the analysis of molecular networking revealed the presence of cyclodipeptides, and compound **1** was produced only under mBHI fermentation condition. Moreover, bioinformatic analysis suggested that compound **1** was closely related to four genes, namely *jat*A–D, encoding core non-ribosomal peptide synthetase and acetyltransferase.

## 1. Introduction

Deep-sea-derived microorganisms may produce uniquely metabolic mechanisms as a result of surviving in extreme conditions of higher pressure, darkness, lower temperature and lack of oxygen, and it was thought to be as a rich source of structurally diverse and bioactive metabolites for drug discovery [1,2,3]. Some compounds derived from deep-sea bacteria exhibit significant biological properties, such as antimicrobial [4], anti-inflammatory [5], antioxidant [6], antiviral [7], and cytotoxic [8] activities. The genus *Janibacter* that has been isolated from various environmental sources [9] possesses strong biodegradation abilities for polycyclic aromatic hydrocarbons [10], pentachlorophenol [11], dibenzofuran [12], mono-chlorinated dibenzo-*p*-dioxins [13] and crude petroleum oil [14], but few metabolites from the genus are reported.

**Figure 1:**
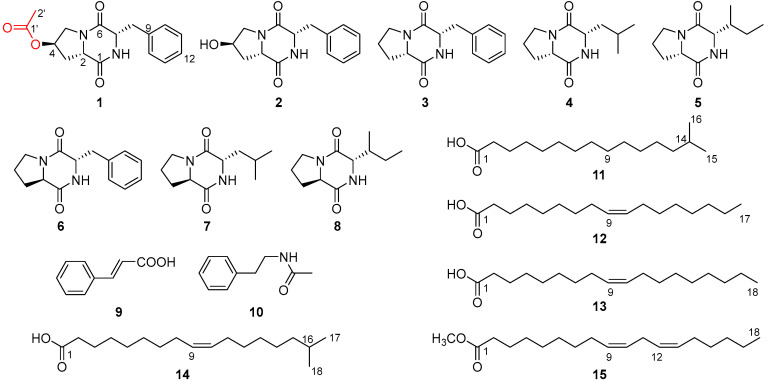
The chemical structures of compounds **1**–**15**.

As a continuous effort in finding novel metabolites with structural diversity and biological potentiality from bacteria isolated from the deep-sea sediment, we systematically investigate the chemical constituents and bioinformation of SCSIO 52865 collected from the South China Sea at a depth of 3448 m. The eleven different media were used for cultivating the strain based on the OSMAC (One Strain Many Compounds) strategy [15]. Together with the analysis of molecular networking [16], the mBHI medium was selected as fermentation condition in large scale due to its widespread nodes distributing in molecular networking clusters. The strategy seems to be a simple and efficient for awakening silent biosynthetic gene clusters, and we have reported some new compounds in our previous work [17,18]. Eventually, the EtOAc extract was obtained from a total of 47 L fermentation broth using Erlenmeyer flasks shaking. One new diketopiperazine, janibatide A (**1**), along with fourteen known compounds (**2**–**15**), was further isolated and identified (Figure 1). The planar structure of **1** was elucidated by detailed 1D/2D NMR spectroscopy and HRESIMS data, and its absolute configuration was further confirmed by Marfey’s method.

## 2. Results and Discussion

### 2.1. Analysis of Molecular Networking

**Figure 2:**
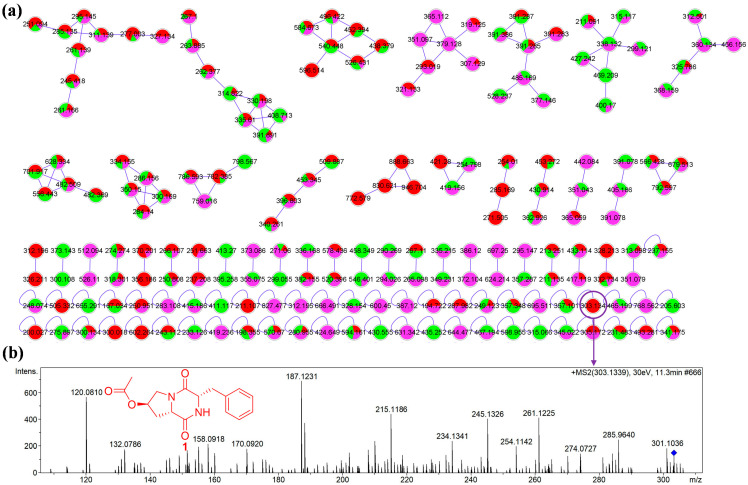
The molecular networking from SCSIO 52865. (**a**) Partial cluster-node graph of molecular networking from three media, in which red, green and pink nodes represented ion peaks from EtOAc extracts of mBHI, mA and mISP2, respectively. The label reparented for parent mass. (**b**) The annotation of a node of parent ion at *m*/*z* 303.134 (the small blue quadrilateral) from molecular networking was corresponding to the chemical structure of compound **1**.

To fully uncover the metabolic profiles of SCSIO 52865 isolated from deep-sea sediment, eleven different media (Table S1) were used to explore for metabolites based on OSMAC strategy. In aggregate, except for mM20, other HPLC chromatography (Figure S1) of EtOAc extracts exhibited similar HPLC-UV profiles within the scope of 0~12 min. Three EtOAc extracts from mBHI, mA and mISP2 media were selected to further measure their LC-MS/MS spectra under the positive ESI mode and the MS/MS data was used to construct molecular networking (MN), due to slightly different HPLC-UV profiles 12 min later. A visual graph (Figure 2a and Figure S2) was drawn using Cytoscape software [19], in which red, green and pink nodes represented for MS data from EtOAc extracts of mBHI, mA and mISP2 fermentation broths, respectively. The MN contained eighteen network clusters within >two nodes and twenty-three network clusters within two nodes. In addition, we found that the above three EtOAc extracts could produce the same parent mass or structural analogues in MN clusters, and simultaneously they were alone able to generate characteristic parent mass in single nodes. These results were coincident with those of HPLC chromatography (Figure S1). Additionally, we discovered that the red nodes, corresponding to EtOAc extracts from mBHI fermentation broth, widely distributed in different clusters and possessed some unique parent mass. Consequently, the mBHI medium was used as fermentation condition on a large scale for enough samples. Furthermore, a particular node of parent ion at *m*/*z* 303.134 in the MN (Figure 2b) was corresponding to compound **1**, a new natural cyclic dipeptide derivative, whose planar structure was recorded on a patent of solid phase apparatus, but without detailed NMR data [20]. Meanwhile, some known cyclodipeptides were further illustrated in the MN (Figure S2), corresponding to the characteristic signals appeared in HPLC-UV chromatography, which possessed terminal absorption profiles with major polarity.

### 2.2. Structural Elucidation of Compounds

**Figure 3:**
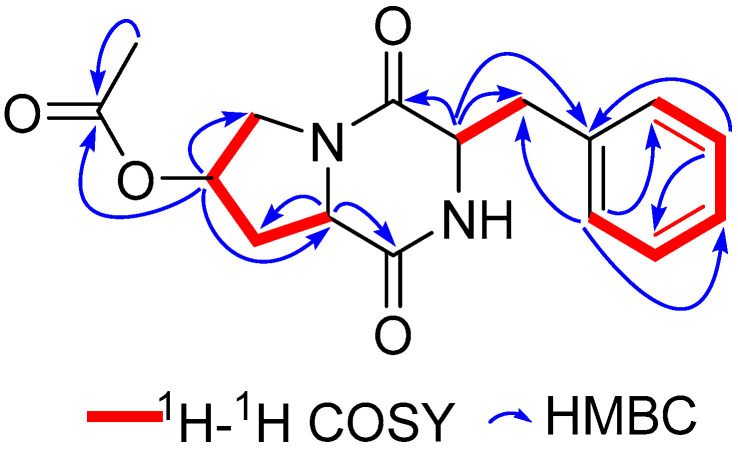
Key ^1^H-^1^H COSY and HMBC correlations of compound **1**.

Compound **1** was isolated as a white powder. Its molecular formula was assigned as C_16_H_18_N_2_O_4_ based on the molecular ion peak at *m*/*z* 303.1348 [M+H]^+^ (calcd for C_16_H_19_N_2_O_4_ 303.1339), indicating nine degrees of unsaturation. The ^1^H NMR spectrum (Figure S7, Table 1) of **1** displayed five olefinic protons at *δ*_H_ 7.29 (H-11, 13, overlapped) and *δ*_H_ 7.24 (H-10, 12, 14, overlapped), five oxygenated or nitrogenated protons at *δ*_H_ 5.13 (H-4, t, *J* = 5.4 Hz), *δ*_H_ 4.49 (H-7, td, *J* = 5.1, 1.8 Hz), *δ*_H_ 4.34 (H-2, ddd, *J* = 11.8, 5.9, 1.9 Hz), *δ*_H_ 3.89 (H-5a, dd, *J* = 13.8, 5.6 Hz), *δ*_H_ 3.38 (H-5b, d, *J* = 13.8 Hz), one methylene proton at *δ*_H_ 3.17 (H-8, m), and one methyl proton at *δ*_H_ 2.02 (H-2′, s). The ^13^C and DEPT spectra (Figure S8, Table 1) showed sixteen resonances for four quaternary carbons (three carbonyl carbons at *δ*_C_ 172.0, *δ*_C_ 170.5, and *δ*_C_ 167.0; one olefinic carbon at *δ*_C_ 137.0), eight methines (five olefinic carbons at *δ*_C_ 131.1 × 2, *δ*_C_ 129.6 × 2, and *δ*_C_ 128.2; one oxygenated carbon at *δ*_C_ 71.0; and two nitrogenated carbons at *δ*_C_ 58.4 and *δ*_C_ 57.7), three methylenes (one nitrogenated carbon at *δ*_C_ 52.9; and two carbons at *δ*_C_ 38.2 and *δ*_C_ 36.1), and one methyl (*δ*_C_ 20.9). The above-mentioned data suggested the presences of two structural fragments for benzyl and acetyl moieties, which were confirmed by detailed analyses of HMBC and ^1^H-^1^H COSY correlations (Figure 3), and we further determined the structure of cyclo(Pro-Phe) scaffold based on both the similar NMR data with cyclo(L-*trans*-Hyp-L-Phe) (compound **2**) [21] and 2D NMR spectra (Figures S9–S12). The acetyl moiety was positioned at C-4 according to HMBC correction from H-4 to C-1′, to establish the planar structure of **1**.

By comparing specific ration data of **1** ([*α*]${}_{D}^{25}$ −22.8) with those of **2** ([*α*]${}_{D}^{25}$ −33.1), compound **1** was elucidated as cyclo(4*R*-acetyl-L-Pro-L-Phe), which was strongly supported by the result of Marfey’s analysis. The absolute configurations of 4-hydroxy-Pro and Phe moieties were unambiguously determined as 4*R*-hydroxy-L-Pro and L-Phe by comparison with the HPLC retention times of standard amino acid derivatives (Table 2), in which 4*R*-hydroxy-L-Pro-L-FDAA derivative was derived from cyclo(L-*trans*-Hyp-L-Leu) that was determined by single-crystal X-ray diffraction analysis in our previous paper [17]. Thus, the structure of compound **1** was confirmed and named as janibatide A.

Compounds **2**–**8** were, respectively assigned as cyclo(L-*trans*-Hyp-L-Phe) [21], cyclo(L-Pro-L-Phe) [17], cyclo(L-Pro-L-Leu) [17], cyclo(L-Pro-L-Ile) [22], cyclo(D-Pro-L-Phe) [21], cyclo(D-Pro-L-Leu) [23] and cyclo(D-Pro-L-Ile) [17] by comparison of their NMR data (Tables S5–S8) and specific rotations with those values reported in the literature, then compounds **1**, **2**, **5**–**7** were further verified using Marfey’s analysis. Compounds **9** and **10** were, respectively, elucidated as *trans*-cinnamic acid [24] and *N*-phenethylacetamide [25] by comparison of their NMR data (Table S9) with those described in the literature. Moreover, compounds **11**–**15** were determined as fatty acids, including 14-methylpentadecanoic acid (**11**) [26], (*Z*)-heptadec-9-enoic acid (**12**) [27], oleic acid (**13**) [28], (*Z*)-16-methylheptadec-9-enoic acid (**14**) [29] and methyl linoleate (**15**) [30] by comparison of their MS and NMR data (Tables S10–S12) with those reported in the literature, in which compounds **12**–**14** were further confirmed by GC-MS analysis (Figures S15–S18).

### 2.3. Putatively Biosynthetic Pathway of Compound ***1** *

**Figure 4:**
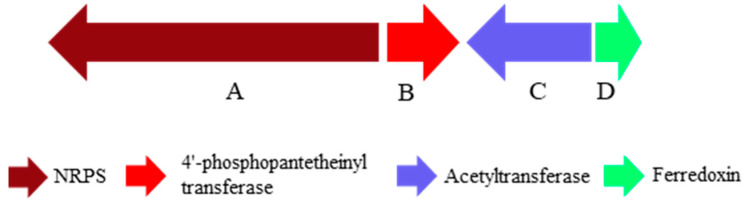
Putative *jat* gene cluster.

**Scheme 1:**
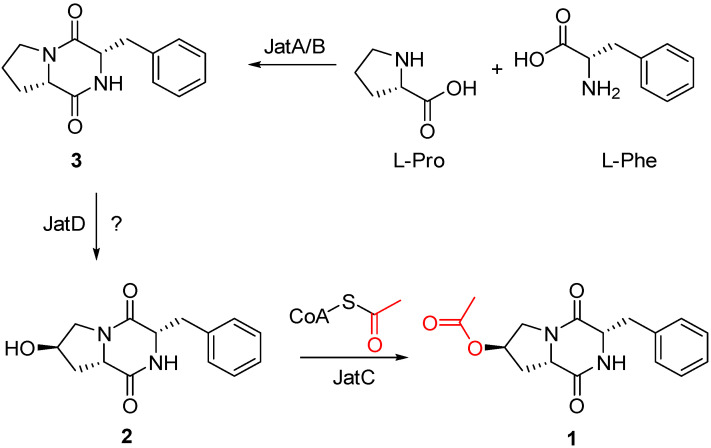
Isolated compounds and proposed biosynthesis pathway for janibatide A (**1**).

A circular contig of 3,495,359 bp (Figure S3) with a GC content of 70.95% was produced by genome sequencing. Additionally, four secondary metabolite biosynthetic gene clusters (BGCs) were predicted by antiSMASH bacterial version 6.1.1 with the default settings [31], namely regions 1.1–1.4, corresponding to T3PKS, ectoine, terpene and NRPS-like types (Table S2 and Figure S4). In general, cyclic dipeptides scaffold can be naturally synthesized by either non-ribosomal peptide synthetases (NRPSs) or CDP synthases (CDPSs) [32]. Clearly, the only NRPS-like-type cluster was likely responsible for biosynthesis of cyclic dipeptide derivatives in the strain. The detailed bioinformation analysis revealed that compound **1** was closely related to four core genes, *jat*A–D (Figure 4, Scheme 1, and Tables S3 and S4).

Firstly, compound **3** was putatively synthesized by a combination of non-ribosomal peptide synthetase (JatA) and 4′-phosphopantetheinyl transferase (JatB). The synergistic enzymes were likely to possess one specific proline recognition domain to form cyclic dipeptide derivatives containing L- or D-proline moiety according to isolated structures. Subsequently, compound **3** was converted to compound **2** by an oxidation reaction putatively catalyzed by the enzyme JatD, a ferredoxin that can transfer electrons and catalyze the formation of hydroxy in corporation with oxygenase, especially P450 [33,34]. However, we did not seek out any oxygenase in near both upstream and downstream of the ferredoxin, possibly suggesting a particularly biosynthetic mechanism that needs to be studied in future. In addition, we discovered that only cyclo(L-Pro-L-Phe) was hydroxylated from all isolated cyclodipeptides; however, others, including cyclo(D-Pro-L-Phe), were not hydroxylated, which indicated that the enzyme JatD probably possessed substrate specificity. Furthermore, compound **1** was putatively produced by a reaction of compound **2** with acetyl-CoA catalyzed by the enzyme JatC. Meanwhile, two putative prolyl-tRNA synthetase genes (proS) were found by Swissprot annotation (Table S3), in connection with cyclodipeptides containing L- or D-proline moiety. Moreover, analysis of structural characteristics revealed that compounds **1**–**8** all possessed proline moiety, and compounds **6**–**8** had the same planar structures with compounds **3**–**5**, respectively; the only difference was the configuration of proline residue. Given the fact that only one NRPS-like gene cluster existed, compounds **4**–**8** were putatively synthesized by JatA/B. The synergistic enzymes exhibited specificity for Pro, the first amino acid, but have not specificity for another amino acid which is likely as hydrophobic amino acids such as Phe, Leu and Ile.

Moreover, the KEGG database annotated the biosynthesis of unsaturated fatty acids (Figure S5), including C16:0, C18:Δ9, and C18:Δ9,12, in accord with the isolated compounds **11**–**15**, freed by the acyl-CoA Thioesterase (TesB [35]) (Table S3).

### 2.4. Biological Activities

All isolated compounds were tested for antibacterial activity against three Gram-positive and one Gram-negative bacteria (*Bacillus subtilis*, *Bacillus thuringiensis*, *Staphylococcus aureus* and *Escherichia coli*), resulting in that none of which exhibited obvious antibacterial activity. Additionally, compound **1** was measured for cytotoxicity against HL-60 human tumor cell line by CCK-8 assay [36], but the result displayed no obvious inhibitory activity at concentration of 100 µM. Moreover, all compounds were evaluated for *α*-glucosidase inhibitory activity, and all of which did not show inhibitory effects at the concentration of 166.7 µg/mL (Table S13). Instead, the fatty acids, especially compound **12**, seemed to significantly increase the conversion of PNPG to PNP catalyzed by *α*-glucosidase in comparison with the negative control.

## 3. Materials and Methods

### 3.1. General Experimental Procedures

Column chromatography (CC): Claricep Flash C-18 Column (20–40 µm, Agela Technologies, Tianjin, China), Sephadex LH-20 (100–200 μm, Pharmacia, Uppsala, Sweden). UV spectra were run on a UV-2600 spectrophotometer (Shimadzu, Kyoto, Japan). IR Spectra were measured on an IR Affinity-1 spectrometer (Shimadzu, Kyoto, Japan). ^1^H, ^13^C and 2D NMR spectra were measured on Bruker AV500 or Bruker AVⅢ HD 700 MHz spectrometer (Bruker, Billerica, MA, USA) using TMS as the internal standard. HRESIMS and LC-MS/MS were recorded on a Bruker maXis quadrupole-time-of-flight mass spectrometer (Bruker, Billerica, MA, USA). MPLC (CHEETAH MP 200, Agela Technologies, Tianjin, China) was equipped with Flash C-18 Column (Agela Technologies, Tianjin, China). HPLC (Agilent 1260) was equipped with YMC-Pack ODS-A (250 × 4.6 mm or 250 × 10.0 mm, 5 μm, YMC, Ishikawa-ken, Japan) column, YMC-Pack Ph (250 × 4.6 mm or 250 × 10.0 mm, 5 μm, YMC, Ishikawa-ken, Japan) column.

### 3.2. Microorganism and Growth Conditions

The strain SCSIO 52865 was isolated and purified from sediment collected from the South China Sea (13°08′40″ N, 114°38′21″ E) at a depth of 3448 m. Analysis of the 16S rRNA sequence revealed that the strain was a member of the *Janibacter* sp. and shared 99.13% identity with *Janibacter cremeus* HR08-44(T) (GenBank accession no. AB778259). Initially, the strain was cultivated in eleven different liquid media (Table S1). Subsequently, the strain was cultivated in 500 mL Erlenmeyer flasks each containing 200 mL of mBHI culture broth at 28 °C for 7 days with shaking rate at 180 rpm, and a total of 47 L fermentation was obtained.

### 3.3. Whole Genome Sequencing and Bioinformatic Analysis

The genome of SCSIO 52865 was extracted by Oxford Nanopore Technologies (ONT) protocol [37]. In brief, the quality of high molecular weight genomic DNA (gDNA) was controlled by a combination of Nanodrop, Qubit and 0.35% agarose gel electrophoresis, and large fragments were selected by automatic BluePippin system. Subsequently, the library was constructed by the SQK-LSK109 ligation kit (Nanopore, Oxford, UK). The circular contig of 3,495,359 bp with a GC content of 70.95% was assembled by using Canu v1.5 for assembly, Racon v3.4.3 for rectification, Circlator v1.5.5 for cyclization and Pilon v1.22 for correction. The genomic sequence has been deposited in GenBank under accession number CP115184. Genomic sequence annotation was performed by using general database, including KEGG, Pfam, and SwissProt, and the putative secondary metabolite BGCs were predicted by antiSMASH version 6.1.1.

### 3.4. Extraction and Isolation

The culture (47 L) was extracted three times with an equal volume of EtOAc at room temperature. The EtOAc layer was separated from the aqueous phase, and it was evaporated in vacuo to give a dry EtOAc extract (8.4 g). The EtOAc extract was separated on MPLC C-18 with gradient MeOH/H_2_O to generate Fr.1–Fr.10. Fr.3 (800 g) was chromatographed on Sephadex LH-20 column eluted with MeOH to afford seven subfractions Fr.3.1–Fr.3.7. Fr.3.3 (700 mg) was again subjected to MPLC C-18 column eluted with gradient MeOH/H_2_O from 15% to 40% to yield six subfractions Fr.3.3.1–Fr.3.3.6. Fr.3.3.2 (73 mg) was further purified by semi-preparative HPLC to afford **1** (1.4 mg) and **10** (1.8 mg). Fr.3.3.1 (400 mg) was repeatedly purified by semi-preparative HPLC to afford **2** (6.1 mg), **3** (5.0 mg), **4** (28.5 mg), **5** (15.0 mg), **6** (22.3 mg), **7** (19.4 mg) and **8** (5.2 mg). Fr.3.4 (60 mg) was chromatographed on C-18 column, and was further purified by semi-preparative HPLC to afford **9** (2.0 mg). Fr.10 (900 mg) was subjected to Sephadex LH-20 column eluted with MeOH to afford ten subfractions Fr.10.1–Fr.10.10. Fr.10.7 (600 mg) was repeatedly purified by semi-preparative HPLC to afford **11** (52.1 mg), **12** (60.5 mg), **13** (41.2 mg), **14** (10.3 mg), and **15** (1.0 mg).

Janibatide A (**1**): white powder, [*α*]${}_{D}^{25}$ −22.8 (c 0.1, MeOH), UV (MeOH) *λ*_max_ (log *ε*) 206 (3.72) nm. IR (film) *ν*_max_ 3337, 2949, 2835, 1651, 1448, 1410, 1111, 1016, and 675 cm^−1^. HRESIMS *m*/*z* 303.1348 [M+H]^+^ (calcd for C_16_H_19_N_2_O_4_ 303.1339). ^1^H NMR (CD_3_OD, 500 MHz) and ^13^C NMR (CD_3_OD, 126 MHz) (see Table 1 and Figures S7–S12).

Cyclo(L-*trans*-Hyp-L-Phe) (**2**), white powder, ESIMS *m*/*z* 261.6 [M+H]^+^, C_14_H_16_N_2_O_3_, [*α*]${}_{D}^{25}$ −33.1 (c 0.1, MeOH), ^1^H NMR (CD_3_OD, 700 MHz) and ^13^C NMR (CD_3_OD, 176 MHz) (see Table S5).

Cyclo(L-Pro-L-Phe) (**3**), white powder, ESIMS *m*/*z* 245.5 [M+H]^+^, C_14_H_16_N_2_O_2_, [*α*]${}_{D}^{25}$ −95.0 (c 0.1, MeOH), ^1^H NMR (CD_3_OD, 500 MHz) and ^13^C NMR (CD_3_OD, 126 MHz) (see Table S5).

Cyclo(L-Pro-L-Leu) (**4**), white powder, ESIMS *m*/*z* 211.4 [M+H]^+^, C_11_H_18_N_2_O_2_, [*α*]${}_{D}^{25}$ −122.7 (c 0.1, MeOH), ^1^H NMR (CD_3_OD, 500 MHz) and ^13^C NMR (CD_3_OD, 126 MHz) (see Table S6).

Cyclo(L-Pro-L-Ile) (**5**), white powder, ESIMS *m*/*z* 211.4 [M+H]^+^, C_11_H_18_N_2_O_2_, [*α*]${}_{D}^{25}$ −47.9 (c 0.1, MeOH), ^1^H NMR (CD_3_OD, 500 MHz) and ^13^C NMR (CD_3_OD, 126 MHz) (see Table S6).

Cyclo(D-Pro-L-Phe) (**6**), white powder, ESIMS *m*/*z* 245.5 [M+H]^+^, C_14_H_16_N_2_O_2_, [*α*]${}_{D}^{25}$ +65.0 (c 0.1, MeOH), ^1^H NMR (CD_3_OD, 500 MHz) and ^13^C NMR (CD_3_OD, 126 MHz) (see Table S7).

Cyclo(D-Pro-L-Leu) (**7**), white powder, ESIMS *m*/*z* 211.4 [M+H]^+^, C_11_H_18_N_2_O_2_, [*α*]${}_{D}^{25}$ +36.2 (c 0.1, MeOH), ^1^H NMR (CD_3_OD, 500 MHz) and ^13^C NMR (CD_3_OD, 126 MHz) (see Table S8).

Cyclo(D-Pro-L-Ile) (**8**), white powder, ESIMS *m*/*z* 211.4 [M+H]^+^, C_11_H_18_N_2_O_2_, [*α*]${}_{D}^{25}$ +37.2 (c 0.1, MeOH), ^1^H NMR (CD_3_OD, 500 MHz) and ^13^C NMR (CD_3_OD, 126 MHz) (see Table S8).

*trans*-Cinnamic acid (**9**), white powder, ESIMS *m*/*z* 149.2 [M+H]^+^, C_9_H_8_O_2_, ^1^H NMR (CD_3_OD, 700 MHz) and ^13^C NMR (CD_3_OD, 176 MHz), see Table S9.

*N*-Phenethylacetamide (**10**), white powder, ESIMS *m*/*z* 164.2 [M+H]^+^, C_10_H_13_NO, ^1^H NMR (CD_3_OD, 700 MHz) and ^13^C NMR (CD_3_OD, 176 MHz) (see Table S9).

14-Methylpentadecanoic acid (**11**), colorless oil, ESIMS *m*/*z* 255.6 [M−H]^−^, C_16_H_32_O_2_, ^1^H NMR (CDCl_3_, 500 MHz) and ^13^C NMR (CDCl_3_, 126 MHz) (see Table S10).

(*Z*)-Heptadec-9-enoic acid (**12**), colorless oil, ESIMS *m*/*z* 267.7 [M−H]^−^, C_17_H_32_O_2_, GC-MS (see Figure S15), ^1^H NMR (CDCl_3_, 500 MHz) and ^13^C NMR (CDCl_3_, 126 MHz) (see Table S11).

Oleic acid (**13**), colorless oil, ESIMS *m*/*z* 281.7 [M−H]^−^, C_18_H_34_O_2_, GC-MS (see Figure S16), ^1^H NMR (CDCl_3_, 500 MHz) and ^13^C NMR (CDCl_3_, 126 MHz) (see Table S11).

(*Z*)-16-Methylheptadec-9-enoic acid (**14**), colorless oil, ESIMS *m*/*z* 281.7 [M−H]^−^, C_18_H_34_O_2_, GC-MS, see Figure S17, ^1^H NMR (CDCl_3_, 500 MHz) and ^13^C NMR (CDCl_3_, 126 MHz) (see Table S12).

Methyl linoleate (**15**), colorless oil, ESIMS *m*/*z* 295.9 [M+H]^+^, C_19_H_34_O_2_, GC-MS, see Figure S18, ^1^H NMR (CDCl_3_, 700 MHz) and ^13^C NMR (CDCl_3_, 176 MHz) (see Table S12).

### 3.5. Molecular Networking

The experimental procedure has been described in our previous paper [17].

### 3.6. Marfey’s Analysis

Briefly, 0.25 mg amounts of compounds **1**, **2**, **5**–**7**, and cyclo(L-*trans*-Hyp-L-Leu) were hydrolyzed with 6 M HCl (1 mL) at 110 °C for 20 h, and each reaction mixture was cooled to room temperature and evaporated to dryness. The residue was diluted with 100 µL of water and then was treated with 80 µL of 1% acetone solution of L-FDAA and 40 µL of 1 M NaHCO_3_ at 40 °C for 1 h. Then, the reaction was quenched by the addition of 2 M HCl (20 µL), followed by vaporization in vacuo and then dilution with MeOH (200 µL). After filtration through a 0.22 µm syringe filter, a 25 µL aliquot was injected into HPLC for detecting retention times under analytical conditions as follows: column: YMC-Pack Ph (250 × 4.6 mm, 5 µm); A phase: ultrapure water; B phase: CH_3_CN; C phase: aqueous solution of 0.1% formic acid; gradient program: 0 min (60%A–20%B–20%C) to 20 min (52%A–28%B–20%C) to 50 min (37%A–43%B–20%C) to 53 min (37%A–43%B–20%C); flow rate: 1 mL/min; detection: UV 340 nm.

### 3.7. GC-MS Analysis

GC-MS analysis was conducted on GCMS-QP2010 Ultra system (Shimadzu). A 1 µL aliquot (2 mg/mL, dissolved in dichloromethane) was injected into analytic system fitted with RXI-5MS (30.0 m × 0.25 mm, 0.25 µm, Shimadzu, Kyoto, Japan) capillary column. Ultra-high-purity helium was used as carrier gas at a constant flow rate of 1.2 mL/min. The injection, transfer line, and ion source temperatures were 250 °C, 280 °C and 220 °C, respectively. The oven temperature was programmed from 50 °C (hold for 3 min) to 320 °C (hold for 5 min) at a rate of 10 °C/min. The first 4 min was the solvent delay, and the mass spectral data were collected from *m*/*z* 45–500 for 35 min. These fatty acids were further identified by comparison of their mass spectra with those of reference compounds recorded in the National Institute of Standards and Technology (NIST) mass spectral library.

### 3.8. Biological Assays

Antibacterial evaluation against four indicator bacteria was described in our previous work [17].

Cytotoxicity assay was implemented against the human tumor cell line HL-60 using WST-8 reagent. In brief, when the density of HL-60 cell was near 1 × 10^6^/mL, the HL-60 cell was incubated by adding fresh medium for subculture to maintain 4 × 10^5^/mL density. Then, the compound **1** or Staurosporine as positive control in a gradient descent manner was added in well which has added 5 × 10^5^/mL HL-60 with 50 µL, and the plates were incubated in a 5% CO_2_ incubator at 37 °C for 72 h. Subsequently, each cell was treated with 10 µL CCK-8 in a 5% CO_2_ incubator at 37 °C for 2 h. Optical density was performed on an EnVision spectrophotometer (PerkinElmer, Waltham, MA, USA) at 450 nm. The inhibition rate was calculated as the equivalent of the following:

inhibition rate% = [1 − (OD_S_ − OD_BLK_)/(OD_NC_ − OD_BLK_)] × 100% (1)

in which OD_S_, OD_NC_ and OD_BLK_ were the absorption values of well with the additional test compound, and DMSO as the negative control and blank, respectively.

The inhibitory activity of *α*-glucosidase was tested according to the modified method described in the references [38,39], in which *p*-nitrophenyl-*α*-D-glucopyranoside (PNPG) was as a substrate. A total of enzyme solution (20 µL, 0.5 U/mL in 0.2 M PBS, pH 6.8), the tested compound (10 µL, 2 mg/mL in DMSO) and PBS (50 µL) were mixed in a 96-well microplate and preincubated at 37 °C for 15 min. The PNPG solution (20 µL, 5.0 mM in PBS) was added and incubated at 37 °C for 15 min, then Na_2_CO_3_ (20 µL, 0.2 M in PBS) was added to stop the reaction. The released PNP was quantified by a microplate reader (Synergy H1, BioTek, Winooski, VT, USA). Acarbose and DMSO were used as the positive control and negative control, respectively.

## 4. Conclusions

Initially, we used the OSMAC strategy in combination with the visual MN graph to screen for fermentation condition, and we detected some compounds that were not annotated by GNPS, especially compound **1**. Lastly, fifteen compounds were isolated and identified, including one cyclic dipeptide derivative, janibatide A (**1**), from the EtOAc extract via a combination of extensive spectroscopic analyses, Marfey’s method and GC-MS analysis. To our knowledge, this is the first report on the isolation of these metabolites from actinomycete *Janibacter* sp. The bioactive assays showed that all compounds have not displayed obvious antibacterial and cytotoxic activities as well as inhibitory effect against *α*-glucosidase. However, cyclo(L-Pro-L-Phe) (**3**) was reported against *Pencillium expansum* at 2 µg/mL [23], and unsaturated fatty acids, especially oleic acid (**13**), presented modulatory effects in a widely physiological functions [40]. Meanwhile, cyclodipeptides were putatively correlated with quorum-sensing [32], suggesting noncompetitive property, but these remain to be investigated in depth. Moreover, the whole-genome sequencing result indicated that SCSIO 52865 possessed 3.49 Mbp sequence, and only four BGCs were predicted by using antiSMASH platform, in which only NRPS-like type BGCs had a lower similarity to known clusters with value of 7%, and others were beyond 66%. Furthermore, the bioinformatic analysis disclosed that cyclodipeptides (**1**–**8**) were bound up with NRPS-like-type BGCs, and four core genes, *jat*A–D, were putatively responsible for biosynthesis of compound **1**.

## Figures and Tables

**Table 1:** ^1^H and ^13^C NMR data of **1** in methanol-*d*_4_, (TMS, *δ* in ppm, *J* in Hz).

Position	*δ*_H_	*δ*_C_
1		170.5, s
2	4.34 (ddd, 11.8, 5.9, 1.9)	58.4, d
3	2.18 (dd, 13.7, 6.0), 1.45 (ddd, 13.7, 11.8, 5.3)	36.1, t
4	5.13 (t, 5.4)	71.0, d
5	3.89 (dd, 13.8, 5.6), 3.38 (d, 13.8)	52.9, t
6		167.0, s
7	4.49 (td, 5.1, 1.8)	57.7, d
8	3.17 (m)	38.2, t
9		137.0, s
10, 14	7.24 (overlapped)	131.1, d
11, 13	7.29 (overlapped)	129.6, d
12	7.24 (overlapped)	128.2, d
1′		172.0, s
2′	2.02 (s)	20.9, q

^1^H and ^13^C NMR data were recorded at 500 MHz and 126 MHz, respectively.

**Table 2:** The retention times of Marfey’s derivatives of some compounds.

Amino Acid		Standard	Cyclo(L-trans-Hyp-L-Leu)	1	2	5	6	7
t_RL_^1^	Pro	21.570	10.925 ^4^	10.496 ^4^	10.575 ^4^	21.544		
	Phe	38.816		38.353	38.486		38.037	
	Ile	35.863				35.533		
	Leu	36.490	36.621					36.157
t_RD_ ^2^	Pro	23.835					23.250	23.708
	Phe	44.059						
	Ile	42.390						
	Leu	42.851						
t_R_^3^	L-FDAA	22.463	23.277	22.560	22.699	22.821	22.218	22.747

^1^ The retention times of L-amino acid derivatives. ^2^ The retention times of D-amino acid derivatives. ^3^ The retention time of L-FDAA. ^4^ The retention time of 4(*R*)-OH-Pro derivative.

## Data Availability

Not applicable.

## Supplementary Materials

The following supporting information can be downloaded at: https://www.mdpi.com/article/10.3390/molecules28052133/s1, Figure S1: HPLC analysis profiles of EtOAc extracts from eleven media; Figure S2: Integrated cluster-node diagram of molecular networking; Figure S3: Circular genome map of SCSIO 52865; Figure S4: The four secondary metabolite biosynthetic gene clusters from SCSIO 52865; Figure S5: The biosynthesis of unsaturated fatty acid using KEGG annotation; Figure S6–S14: HRESIMS, NMR, HSQC, HMBC, 1H–1H COSY, NOESY, UV, and IR spectra of compound **1**; Figure S15–S18: GC-MS spectra of compounds **12**–**15**; Figure S19: HPLC analysis profiles of Marfey’s derivatives of **1**, **2**, and **5**–**7** at 340 nm; Table S1: Compositions of eleven media; Table S2: The putative secondary metabolite biosynthetic gene clusters from SCSIO 52865 using antiSMASH platform; Table S3: The key genes information from SCSIO 52865; Table S4: Predicted function of the open reading frames; Table S5–S12: NMR data for compounds **2**–**15**; Table S13: Inhibitory effects of compounds **1**–**15**, and acarbose against α-glucosidase.

## References

[1] Carroll A.R., Copp B.R., Davis R.A., Keyzers R.A., Prinsep M.R. (2022). Marine natural products. Nat. Prod. Rep..

[2] Wang Y.N., Meng L.H., Wang B.G. (2020). Progress in research on bioactive secondary metabolites from deep-sea derived microorganisms. Mar. Drugs.

[3] Zhang J., Zhang B., Cai L., Liu L. (2022). New dibenzo-*α*-pyrone derivatives with *α*-glucosidase inhibitory activities from the marine-derived fungus *Alternaria alternata*. Mar. Drugs.

[4] Xu D., Nepal K.K., Chen J., Harmody D., Zhu H., McCarthy P.J., Wright A.E., Wang G. (2018). Nocardiopsistins A-C: New angucyclines with anti-MRSA activity isolated from a marine sponge-derived *Nocardiopsis* sp. HB-J378. Syn. Syst. Biotechnol..

[5] Yan X., Zhou Y.X., Tang X.X., Liu X.X., Yi Z.W., Fang M.J., Wu Z., Jiang F.Q., Qiu Y.K. (2016). Macrolactins from marine-derived *Bacillus subtilis* B5 bacteria as inhibitors of inducible nitric oxide and cytokines expression. Mar. Drugs.

[6] Wagner M., Abdel-Mageed W.M., Ebel R., Bull A.T., Goodfellow M., Fiedler H.P., Jaspars M. (2014). Dermacozines H–J isolated from a deep-sea strain of *Dermacoccus abyssi* from mariana trench sediments. J. Nat. Prod..

[7] Gustafson K., Roman M., Fenical W. (1989). The macrolactins, a novel class of antiviral and cytotoxic macrolides from a deep-sea marine bacterium. J. Am. Chem. Soc..

[8] Hughes C.C., MacMillan J.B., Gaudêncio S.P., Jensen P.R., Fenical W. (2009). The ammosamides: Structures of cell cycle modulators from a marine-derived *Streptomyces* species. Angew. Chem. Int. Ed..

[9] Lim Y.K., Kweon O.J., Kim H.R., Kim T.H., Lee M.K. (2017). First case of bacteremia caused by *Janibacter hoylei*. APMIS.

[10] Yamazoe A., Yagi O., Oyaizu H. (2004). Degradation of polycyclic aromatic hydrocarbons by a newly isolated dibenzofuran-utilizing *Janibacter* sp. strain YY-1. Appl. Microbiol. Biotechnol..

[11] Khessairi A., Fhoula I., Jaouani A., Turki Y., Cherif A., Boudabous A., Hassen A., Ouzari H. (2014). Pentachlorophenol degradation by *Janibacter* sp., a new actinobacterium isolated from saline sediment of Arid Land. Biomed. Res. Int..

[12] Jin S., Zhu T., Xu X., Xu Y. (2006). Biodegradation of dibenzofuran by *Janibacter* terrae strain XJ-1. Curr. Microbiol..

[13] Iwai S., Yamazoe A., Takahashi R., Kurisu F., Yagi O. (2005). Degradation of mono-chlorinated dibenzo-*p*-dioxins by *Janibacter* sp. strain YA isolated from river sediment. Curr. Microbiol..

[14] Ezzat S.M., Ahmed N.A. (2022). Short-term biodegradation of crude petroleum oil in water by photostimulated *Janibacter terrae* strain S1N1. ACS Omega.

[15] Pan R., Bai X., Chen J., Zhang H., Wang H. (2019). Exploring structural diversity of microbe secondary metabolites using OSMAC strategy: A literature review. Front. Microbiol..

[16] Wang M., Carver J.J., Phelan V.V., Sanchez L.M., Garg N., Peng Y., Nguyen D.D., Watrous J., Kapono C.A., Knaan T.L. (2016). Sharing and community curation of mass spectrometry data with Global Natural Products Social Molecular Networking. Nat. Biotechnol..

[17] Ding W., Li Y., Tian X., Chen M., Xiao Z., Chen R., Yin H., Zhang S. (2022). Investigation on metabolites in structural diversity from the deep-sea sediment-derived bacterium *Agrococcus* sp. SCSIO 52902 and their biosynthesis. Mar. Drugs.

[18] Ding W., Li Y., Chen M., Chen R., Tian X., Yin H., Zhang S. (2022). Structures and antitumor activities of ten new and twenty known surfactins from the deep-sea bacterium *Limimaricola* sp. SCSIO 53532. Bioorg. Chem..

[19] Shannon P., Markiel A., Ozier O., Baliga N., Wang J., Ramage D., Amin N., Schwikowski B., Ideker T. (2003). Cytoscape: A software environment for integrated models of biomolecular interaction networks. Genome Res..

[20] Cody D.R., DeWitt S.H.H., Hodges J.C., Kiely J.S., Moos W.H., Pavia M.R., Roth B.D., Schroeder M.C., Stankovic C.J. (1994). Apparatus for Multiple Simultaneous Synthesis. U.S. Patent.

[21] Xiang W.X., Liu Q., Li X.M., Lu C.H., Shen Y.M. (2020). Four pairs of proline-containing cyclic dipeptides from *Nocardiopsis* sp. HT88, an endophytic bacterium of *Mallotus nudiflorus* L. Nat. Prod. Res..

[22] Park A.R., Jeong S.I., Jeon H.W., Kim J., Kim N., Ha M.T., Mannaa M., Kim J., Lee C.W., Min B.S. (2020). A diketopiperazine, *Cyclo*-(L-Pro-L-Ile), derived from *Bacillus thuringiensis* JCK-1233 controls pine wilt disease by elicitation of moderate hypersensitive reaction. Front. Plant. Sci..

[23] Kumar N., Mohandas C., Nambisan B., Kumar D.R.S., Lankalapalli R.S. (2013). Isolation of proline-based cyclic dipeptides from *Bacillus* sp. N strain associated with rhabitid entomopathogenic nematode and its antimicrobial properties. World J. Microbiol. Biotechnol..

[24] Hanai K., Kuwae A., Takai T., Senda H., Kunimoto K.K. (2001). A comparative vibrational and NMR study of *cis*-cinnamic acid polymorphs and *trans*-cinnamic acid. Spectrochim. Acta A Mol. Biomol. Spectrosc..

[25] Lee M.J., Kim G.J., Shin M.S., Moon J., Kim S., Nam J.W., Kang K.S., Choi H. (2021). Chemical investigation of diketopiperazines and *N*-phenethylacetamide isolated from *Aquimarina* sp. MC085 and their effect on TGF-*β*-induced epithelial–mesenchymal transition. Appl. Sci..

[26] Shindo K., Asagi E., Sano A., Hotta E., Minemura N., Mikami K., Tamesada E., Misawa N., Maoka T. (2008). Diapolycopenedioic acid xylosyl esters A, B, and C, novel antioxidative glyco-C_30_-carotenoic acids produced by a new marine bacterium *Rubritalea squalenifaciens*. J. Antibiot..

[27] Bruns H., Thiel V., Voget S., Patzelt D., Daniel R., Wagner-Döbler I., Schulz S. (2013). *N*-Acylated alanine methyl esters (NAMEs) from *Roseovarius tolerans*, structural analogs of quorum-sensing autoinducers, *N*-acylhomoserine lactones. Chem. Biodivers..

[28] Luo J.R., Che Y.H., Su J.S., Yang Z.B., Zhang Y. (2020). Chemical constituents from petroleum ether extract of Yi medicine *Blaps rynchopetera*. Zhong Yao Cai.

[29] Rontani J.F., Zabeti N., Aubert C. (2009). Double bond migration to methylidene positions during electron ionization mass spectrometry of branched monounsaturated fatty acid derivatives. J. Am. Soc. Mass Spectrom..

[30] Yang M.N., Zhang H., Liu J., Li Y., Li W.T., Xia H.L. (2016). Study on chemical constituents from *Phyllanthus urinaria*. Zhong Cao Yao.

[31] Blin K., Shaw S., Kloosterman A.M., Charlop-Powers Z., van Wezel G.P., Medema M.H., Weber T. (2021). antiSMASH 6.0: Improving cluster detection and comparison capabilities. Nucleic Acids Res..

[32] Mishra A.K., Choi J., Choi S.J., Baek K.H. (2017). Cyclodipeptides: An overview of their biosynthesis and biological activity. Molecules.

[33] Zhu Z., Gao X., Song Z., Li C., Lu F., Tanokura M., Qin H.M. (2020). Development of engineered ferredoxin reductase systems for the efficient hydroxylation of steroidal substrates. ACS Sustain. Chem. Eng..

[34] Okubo S., Ena E., Okuda A., Kozone I., Hashimoto J., Nishitsuji Y., Fujie M., Satoh N., Ikeda H., Shin-ya K. (2021). Identification of functional cytochrome P450 and ferredoxin from *Streptomyces* sp. EAS-AB2608 by transcriptional analysis and their heterologous expression. Appl. Microbiol. Biotechnol..

[35] Naggert J., Narasimhan M.L., DeVeaux L., Cho H., Randhawa Z.I., Cronan J.E., Green B.N., Smith S. (1991). Cloning, sequencing, and characterization of *Escherichia coli* thioesterase II. J. Biol. Chem..

[36] Tiwari K., Wavdhane M., Haque S., Govender T., Kruger H.G., Mishra M.K., Chandra R., Tiwari D. (2015). A sensitive WST-8-based bioassay for PEGylated granulocyte colony stimulating factor using the NFS-60 cell line. Pharm. Biol..

[37] Deamer D., Akeson M., Branton D. (2016). Three decades of nanopore sequencing. Nat. Biotechnol..

[38] Feng J., Yang X.W., Wang R.F. (2011). Bio-assay guided isolation and identification of *α*-glucosidase inhibitors from the leaves of *Aquilaria sinensis*. Phytochemistry.

[39] Yang J.B., Tian J.Y., Dai Z., Ye F., Ma S.C., Wang A.G. (2017). *α*-Glucosidase inhibitors extracted from the roots of *Polygonum multiflorum* Thunb. Fitoterapia.

[40] Sales-Campos H., Souza P.R., Peghini B.C., da Silva J.S., Cardoso C.R. (2013). An overview of the modulatory effects of oleic acid in health and disease. Mini-Rev. Med. Chem..

