# Supplementary material for: Investigation on Metabolites in Structure and Biosynthesis from the Deep-Sea Sediment-Derived Actinomycete Janibacter sp. SCSIO 52865
Source: Molecules. 2023 Feb 24;28(5):2133. doi: 10.3390/molecules28052133 (PMC10003874; doi:10.3390/molecules28052133)

Supplementary data for

Investigation on Metabolites in Structure and Biosynthesis from the Deep-Sea Sediment-Derived Actinomycete *Janibacter* sp. SCSIO 52865

Wenping Ding [1], Yanqun Li [1], Xinpeng Tian [1], Zhihui Xiao [1], Ru Li [1], Si Zhang [1,2,*], Hao Yin [1,2,3,*]

[1] *CAS Key Laboratory of Tropical Marine Bio-resources and Ecology, South China Sea Institute of Oceanology, Chinese Academy of Sciences, Guangzhou 510301, China*

[2] *Southern Marine Science and Engineering Guangdong Laboratory (Guangzhou), Guangzhou 511458, China*

[3] *Sanya Institute of Ocean Eco-Environmental Engineering, Sanya 57200, China*

Content

Table S1. Compositions of eleven liquid media 3
Figure S1. HPLC analysis profiles of EtOAc extracts from eleven media 4
Figure S2. Integrated cluster-node diagram of molecular networking 7
Figure S3. Circular genome map of SCSIO 52865 7
Table S2. The putative secondary metabolite biosynthetic gene clusters from SCSIO 52865 using antiSMASH platform 8
Figure S4. The four secondary metabolite biosynthetic gene clusters from SCSIO 52865 8
Table S3. The key genes information from SCSIO 52865 8
Table S4. Predicted function of the open reading frames 9
Figure S5. The biosynthesis of unsaturated fatty acid using KEGG annotation 9
Figure S6. HRESIMS spectrum of compound 1 10
Figure S7. 1H NMR spectrum (CD_3OD, 500 MHz) of compound 1 10
Figure S8. ^{13}C NMR and DEPT spectra (CD_3OD, 126 MHz) of compound 1 11
Figure S9. HSQC spectrum of compound 1 11
Figure S10. HMBC spectrum of compound 1 12
Figure S11. ^1H–^1H COSY spectrum of compound 1 12
Figure S12. NOESY spectrum of compound 1 13
Figure S13. UV spectrum of compound 1 13
Figure S14. IR spectrum of compound 1 14
Table S5. ^1H and ^{13}C NMR data of compounds 2–3 (Methanol-d_4, δ in ppm, J in Hz). 15
Table S6. ^1H and ^{13}C NMR data of compounds 4–5 (Methanol-d_4, δ in ppm, J in Hz). 16
Table S7. ^1H and ^{13}C NMR data of compound 6 (Methanol-d_4, δ in ppm, J in Hz). 17
Table S8. ^1H and ^{13}C NMR data of compounds 7–8 (Methanol-d_4, δ in ppm, J in Hz). 18
Table S9. ^1H and ^{13}C NMR data of compounds 9–10 (Methanol-d_4, δ in ppm, J in Hz). 19
Table S10. ^1H and ^{13}C NMR data of compound 11 (Chloroform-d, δ in ppm, J in Hz). 19
Table S11. ^1H and ^{13}C NMR data of compounds 12–13 (Chloroform-d, δ in ppm, J in Hz). 20

Table S12. ^{1}H and ^{13}C NMR data of compounds 14–15 (Chloroform-*d*, *δ* in ppm, *J* in Hz). 21
Table S13. Inhibitory effects of compounds 1–15, and acarbose against *α*-glucosidase 22
Figure S15. GC-MS spectrum of compound 12 23
Figure S16. GC-MS spectrum of compound 13 24
Figure S17. GC-MS spectrum of compound 14 25
Figure S18. GC-MS spectrum of compound 15 26
Figure S19. HPLC analysis profiles of Marfey's derivatives of 1, 2, and 5–7 at 340 nm 27

Table S1. Compositions of eleven liquid media

Media	Compositions
1 mA	Glucose (10 g/L), Yeast extract (5 g/L), Soluble starch (12 g/L), Bacterial peptone (5 g/L), NaCl (4 g/L), KH_2PO_4 (0.5 g/L), $MgSO_4{\bullet}7H_2O$ (0.5 g/L), $CaCO_3$ (2 g/L), Sea salt (30 g/L).
2 mK	Yeast extract (2 g/L), Bacterial peptone (2 g/L), Glucose (2 g/L), Mannitol (3 g/L), Malt extract (5 g/L), Peptone from soybean (5 g/L), Soluble starch (5 g/L), Sea salt (30 g/L).
3 m2216E	2216E (37.4 g/L), Glucose (15 g/L), Sea salt (30 g/L).
4 mAm2ab	Soluble starch (5 g/L), Peptone from soybean (5 g/L), Glucose (20 g/L), Yeast extract (2 g/L), Bacterial peptone (2 g/L), KH_2PO_4 (1 g/L), $MgSO_4{\bullet}7H_2O$ (0.5 g/L), $CaCO_3$ (2 g/L), Sea salt (30 g/L).
5 mBHI	BHI broth (38.5 g/L), Sea salt (30 g/L).
6 mISP2	Yeast extract (4 g/L), Malt extract (10 g/L), Glucose (4 g/L), Sea salt (30 g/L).
7 mISP4	Soluble starch (10 g/L), KH_2PO_4 (2 g/L), $MgSO_4{\bullet}7H_2O$ (1 g/L), NaCl (1 g/L), $(NH_4)_2SO_4$ (2 g/L), $CaCO_3$ (3 g/L), TS (1 mL/L), Yeast extract (0.5 g/L), Bacterial peptone (1 g/L), Sea salt (30 g/L).
8 mRA	Soluble starch (20 g/L), Glucose (10 g/L), Malt extract (10 g/L), Maltose (10 g/L), Corn steep liquor (5 g/L), $CaCO_3$ (2 g/L), Sea salt (30 g/L).
9 mJNP1A	Soluble starch (20 g/L), Peptone of fish powder (2 g/L), Trehalose Dihydrate (2 g/L), Beef extract (3 g/L), Sea salt (30 g/L).
10 mM20	Soybean flour (15 g/L), Soluble starch (20 g/L), Yeast extract (5 g/L), Bacterial peptone (2 g/L), NaCl (4 g/L), $CaCO_3$ (2 g/L), Sea salt (30 g/L).
11 mMCQ1	Soluble starch (10 g/L), Glucose (10 g/L), Bacterial peptone (5 g/L), Yeast extract (3 g/L), Beef extract (2 g/L), KH_2PO_4 (0.5 g/L), $MgSO_4{\bullet}7H_2O$ (0.5 g/L), KBr (1 g/L), $CaCO_3$ (2 g/L), Sea salt (30 g/L).

TS: Trace salts solution, $FeSO_4{\bullet}7H_2O$ 0.1 g, $MnCl_2{\bullet}4H_2O$ 0.1 g, $ZnSO_4{\bullet}7H_2O$ 0.1 g, in H_2O 100 mL.

Figure S1. HPLC analysis profiles of EtOAc extracts from eleven media

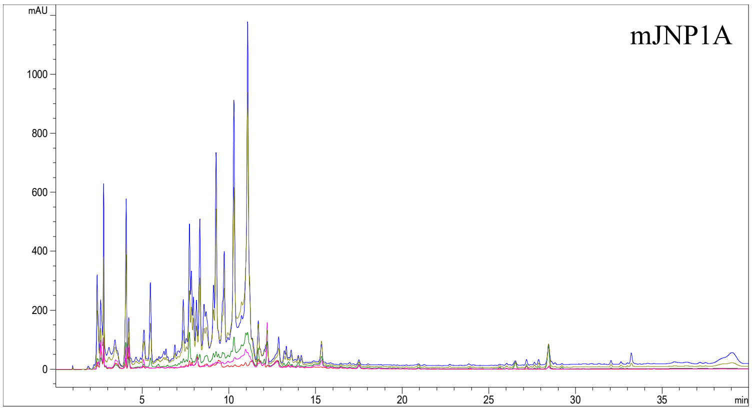

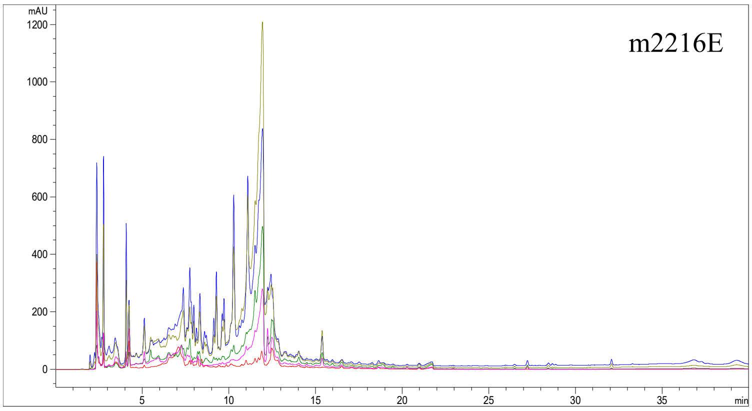

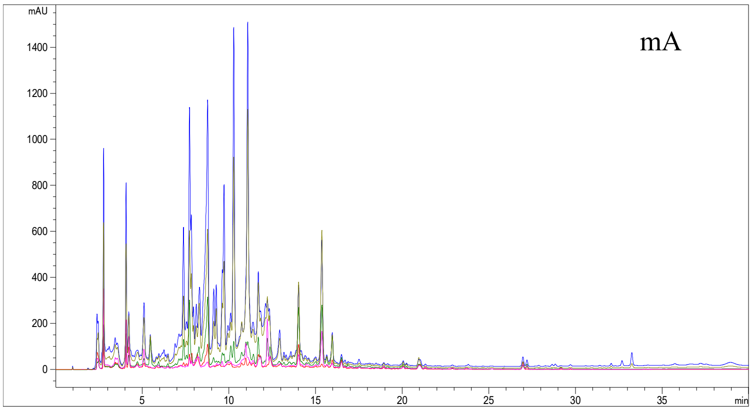

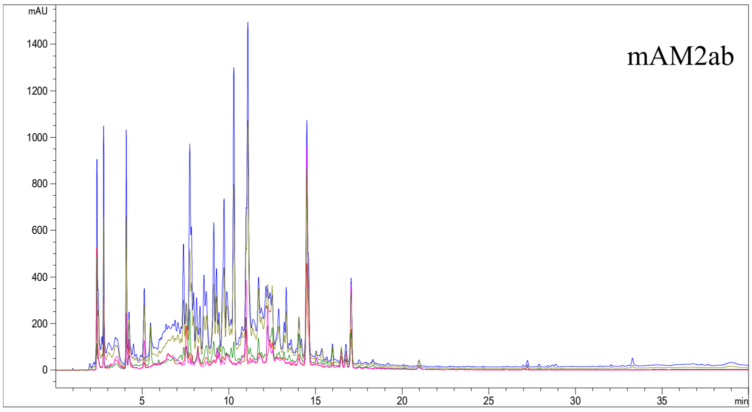

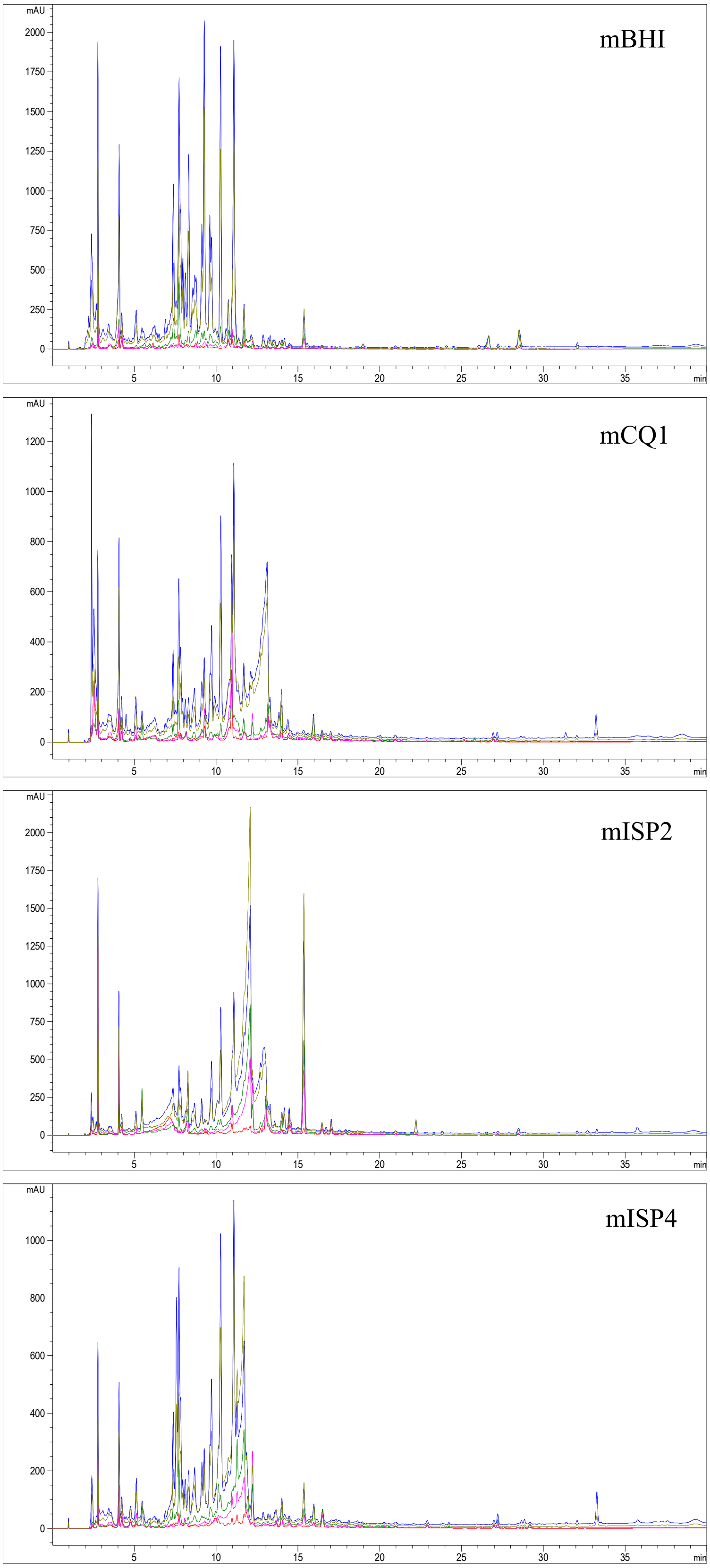
mAU
mBHI
mCQ1
mISP2
mISP4
min

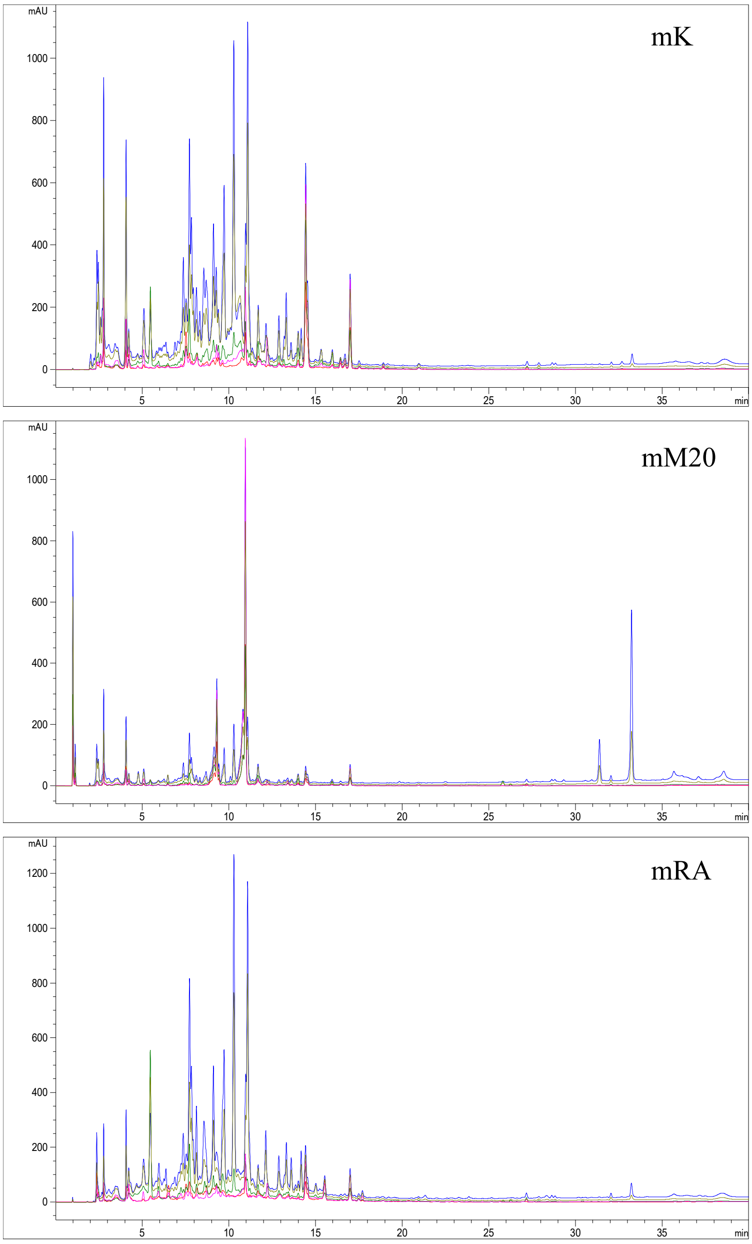

UV detection at 202 nm (blue), 210 nm (yellow), 230 nm (green), 254 nm (pink) and 280 nm (red).

Figure S2. Integrated cluster-node diagram of molecular networking

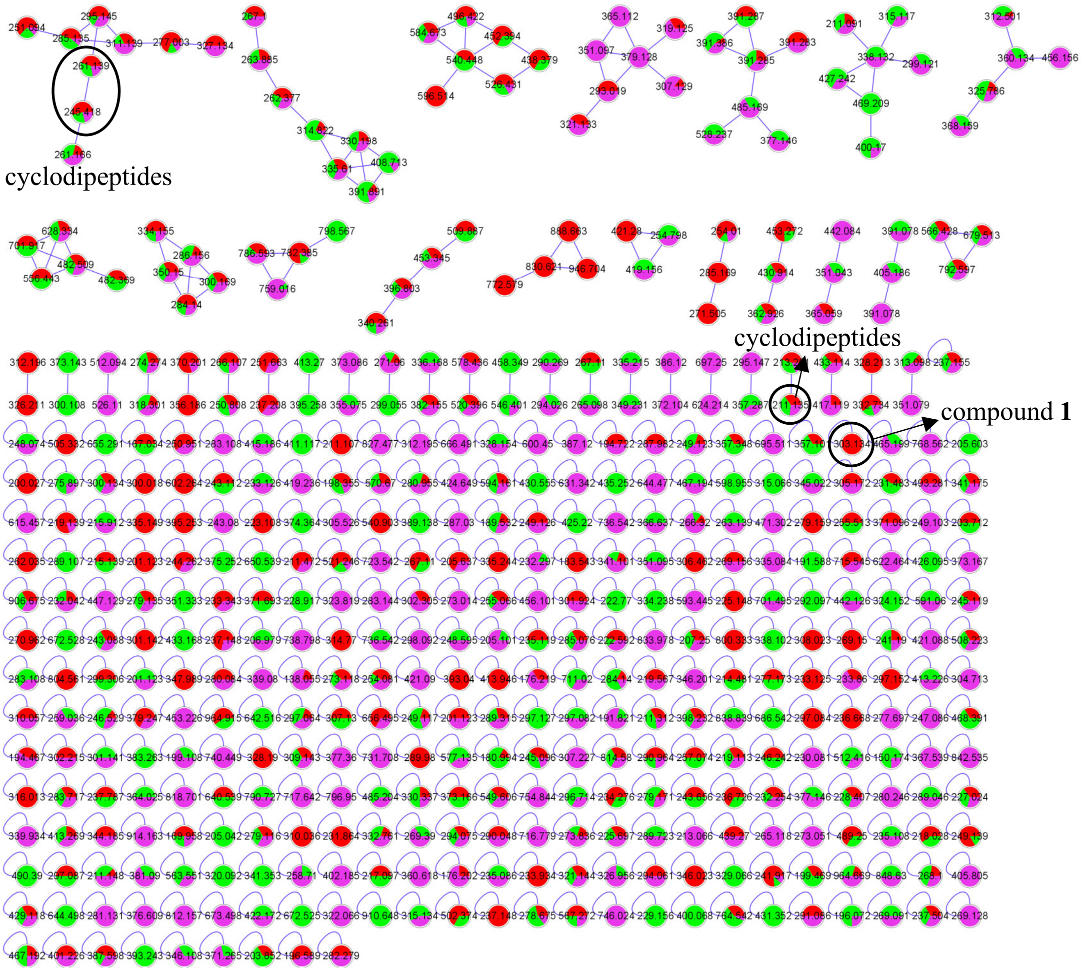

Figure S3. Circular genome map of SCSIO 52865

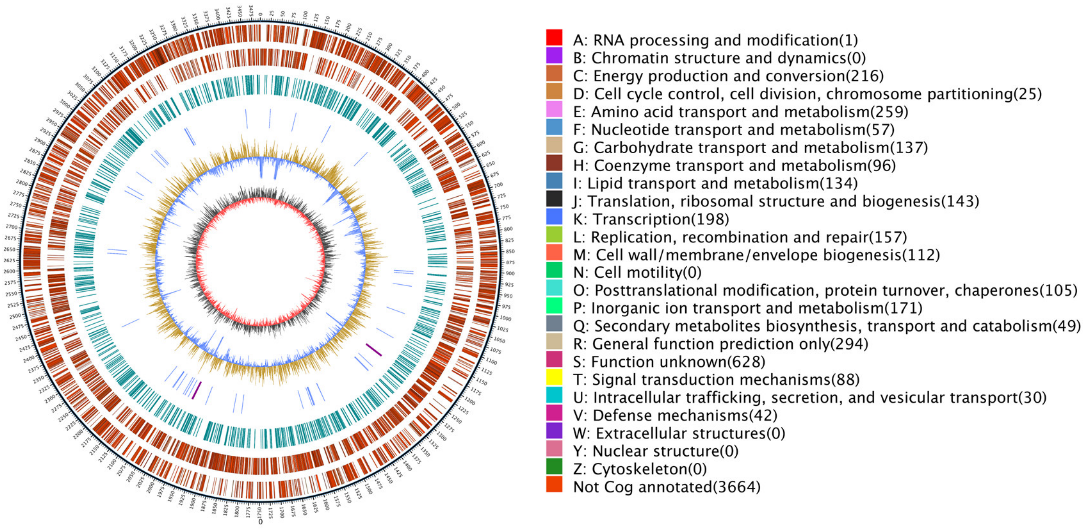

Table S2. The putative secondary metabolite biosynthetic gene clusters from SCSIO 52865 using antiSMASH platform

Regions	Types	Location	Total
1.1	T3PKS	360,465 - 401,553 nt	41,089 nt
1.2	ectoine	971,706 - 982,101 nt	10,396 nt
1.3	terpene	2,030,508 - 2,051,371 nt	20,864 nt
1.4	NPRS-like	3,332,428 - 3,375,034 nt	42,607 nt

Figure S4. The four secondary metabolite biosynthetic gene clusters from SCSIO 52865

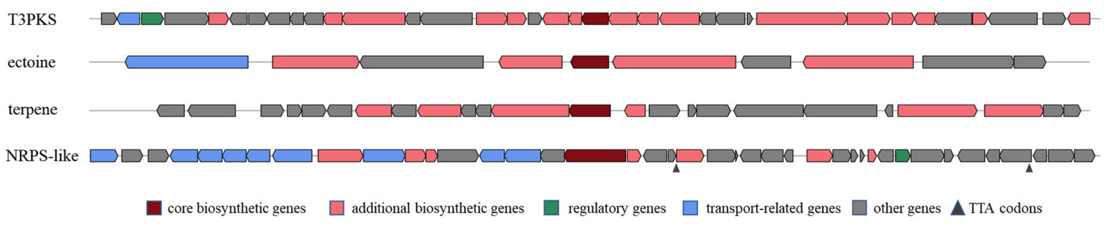

Table S3. The key genes information from SCSIO 52865

Gene	Orientation	Location	Swissprot_target_ID	E-value	Identity	Cover
jatA	(-)	3352428 - 3355034 (2607 nt)	P80574(AROF_STRCO)	3.00E-157	61.24%	94.57%
jatB	(+)	3355098 - 3355670 (573 nt)	Q6AF95(AROB_LEIXX)	1.00E-128	65.72%	99.43%
jatC	(-)	3355770 - 3356753 (984 nt)	staT	6E-04	46.15%	15%
jatD	(+)	3356812 - 3357147 (336 nt)	A0QRD3(MENB_MYCS2)	6.30E-124	74.33%	97.99%
GE001391	(+)	1,522,183 - 1,523,133 (951 nt)	tesB	3.7E-50	40.82%	82.91%
GE000333	(+)	363,533- 365,320 (1788 nt)	proS	1.1E-239	69.7%	99.5%
GE002558	(-)	2,717,282-2,717,749 (468 nt)	proS	1.20E-06	32.61%	59.35%

Table S4. Predicted function of the open reading frames

Protein	Amino Acids (AA)	Putative Function	Homolog (Enzyme, Origin)	AA cover/ identity	Ref.
JatA	869	Non-ribosomal peptide synthetase	PltF [*Pseudomonas protegens*]	40%/30.93%	Q4KCY5.1
JatB	191	4'-phosphopantetheinyl transferase	hypothetical protein [*Actinomadura atramentaria*]	78%/41.71%	WP_019630033.1
JatC	328	Acetyltransferase	hypothetical protein [*Marihabitans asiaticum*]	93%/56.03%	WP_170236173.1
JatD	112	Ferredoxin	FdxA [*Mycobacterium smegmatis*]	95%/74.53%	P00215.1

Figure S5. The biosynthesis of unsaturated fatty acid using KEGG annotation

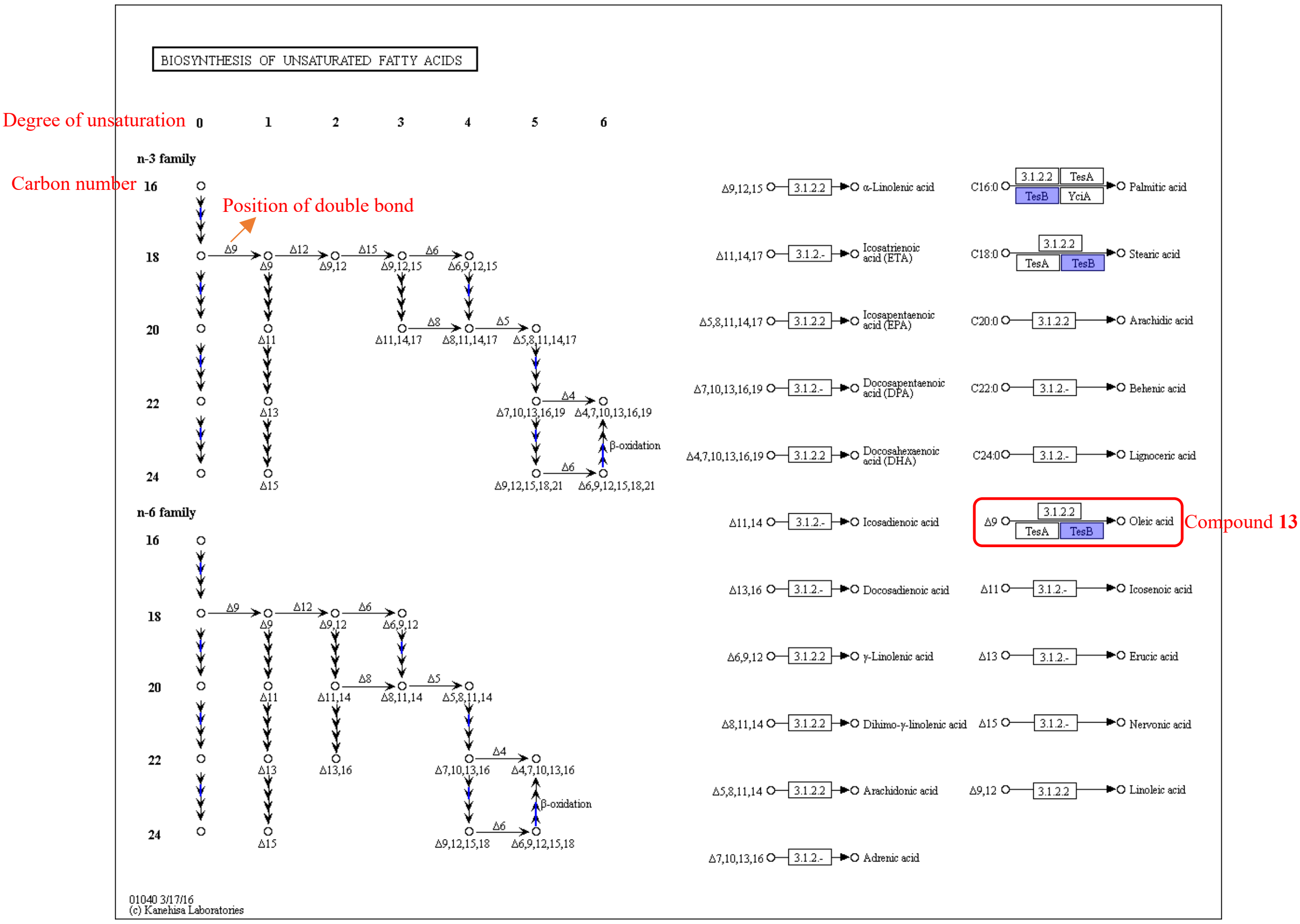

TesB (highlighted in blue): Acyl-CoA thioesterase II. n-3 or n-6 family: a family of fatty acids has a double bond between C-3 and C-4, or C-6 and C-7, counting from the terminal methyl.

Figure S6. HRESIMS spectrum of compound **1**

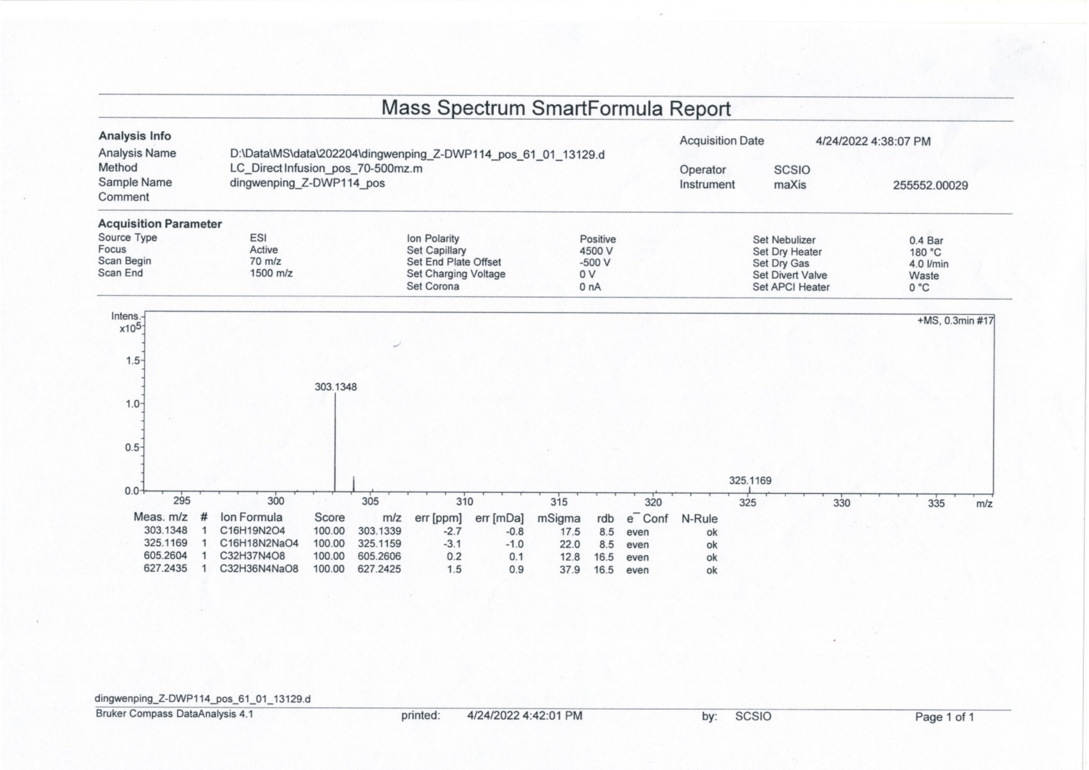
Mass Spectrum SmartFormula Report

Analysis Info

Analysis Name D:\Data\MS\data\202204\dingwenping_Z-DWP114_pos_61_01_13129.d
Method LC_Direct Infusion_pos_70-500mz.m
Sample Name dingwenping_Z-DWP114_pos
Comment

Acquisition Date 4/24/2022 4:38:07 PM
Operator SCSIO
Instrument maXis 255552.00029

Acquisition Parameter

Source Type	ESI	Ion Polarity	Positive	Set Nebulizer	0.4 Bar
Focus	Active	Set Capillary	4500 V	Set Dry Heater	180 °C
Scan Begin	70 m/z	Set End Plate Offset	-500 V	Set Dry Gas	4.0 l/min
Scan End	1500 m/z	Set Charging Voltage	0 V	Set Divert Valve	Waste
		Set Corona	0 nA	Set APCI Heater	0 °C

Meas. m/z	#	Ion Formula	Score	m/z	err [ppm]	err [mDa]	mSigma	rdb	e¯ Conf	N-Rule
303.1348	1	C16H19N2O4	100.00	303.1339	-2.7	-0.8	17.5	8.5	even	ok
325.1169	1	C16H18N2NaO4	100.00	325.1159	-3.1	-1.0	22.0	8.5	even	ok
605.2604	1	C32H37N4O8	100.00	605.2606	0.2	0.1	12.8	16.5	even	ok
627.2435	1	C32H36N4NaO8	100.00	627.2425	1.5	0.9	37.9	16.5	even	ok

dingwenping_Z-DWP114_pos_61_01_13129.d
Bruker Compass DataAnalysis 4.1 printed: 4/24/2022 4:42:01 PM by: SCSIO Page 1 of 1

Figure S7. 1H NMR spectrum (CD_3OD, 500 MHz) of compound **1**

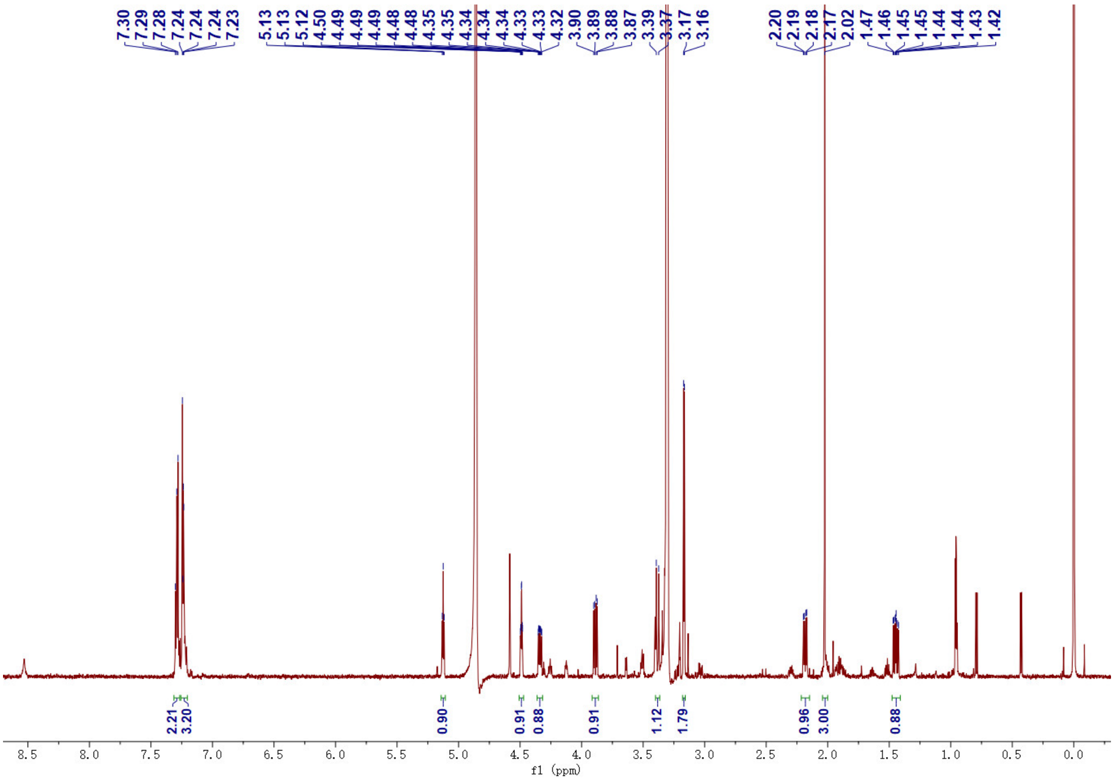

Figure S8. ^{13}C NMR and DEPT spectra (CD_3OD, 126 MHz) of compound **1**

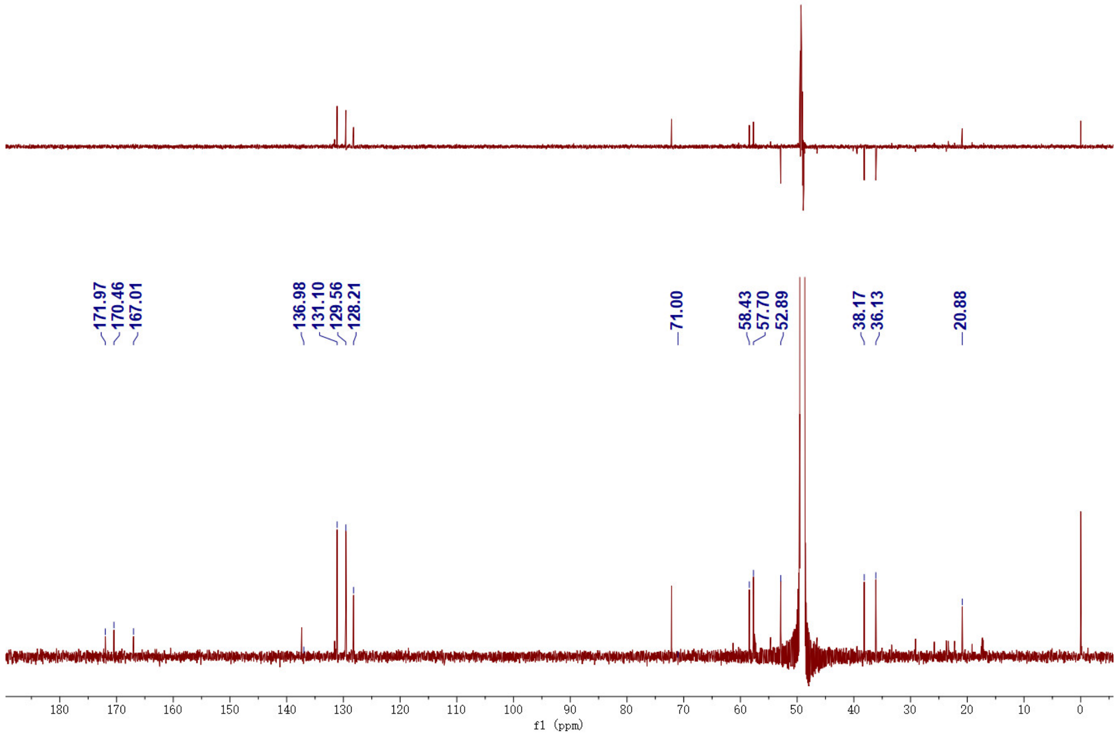

Figure S9. HSQC spectrum of compound **1**

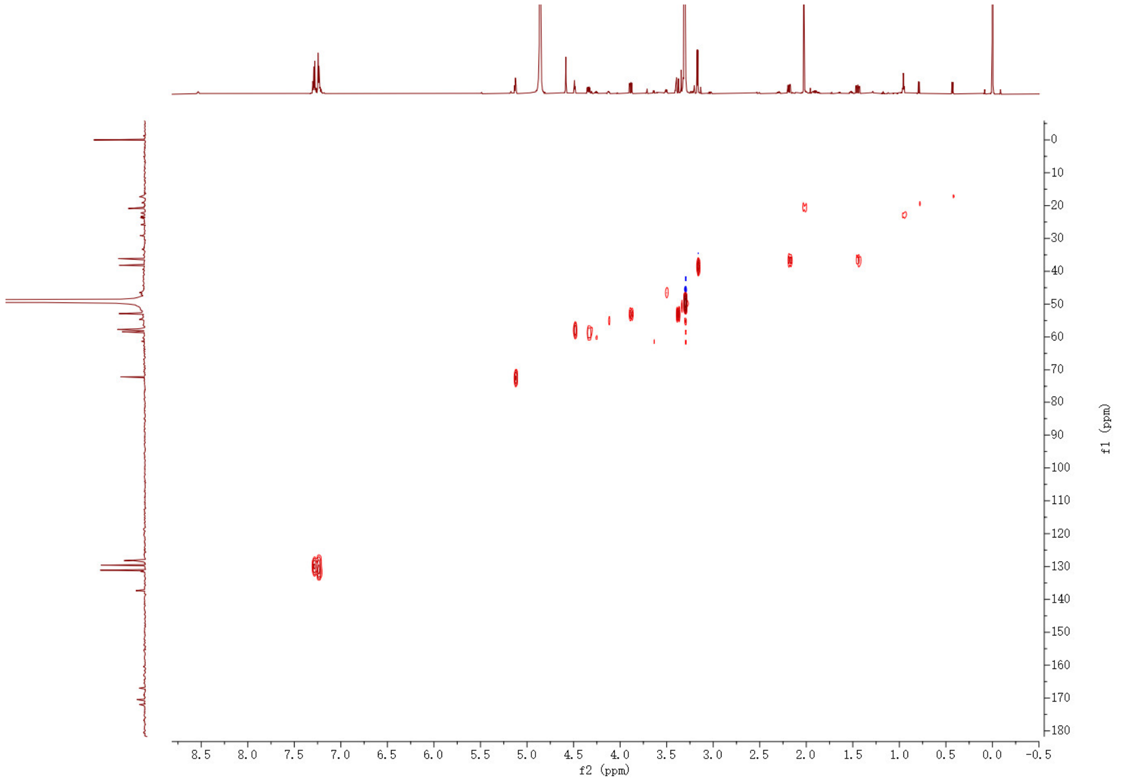

Figure S10. HMBC spectrum of compound **1**

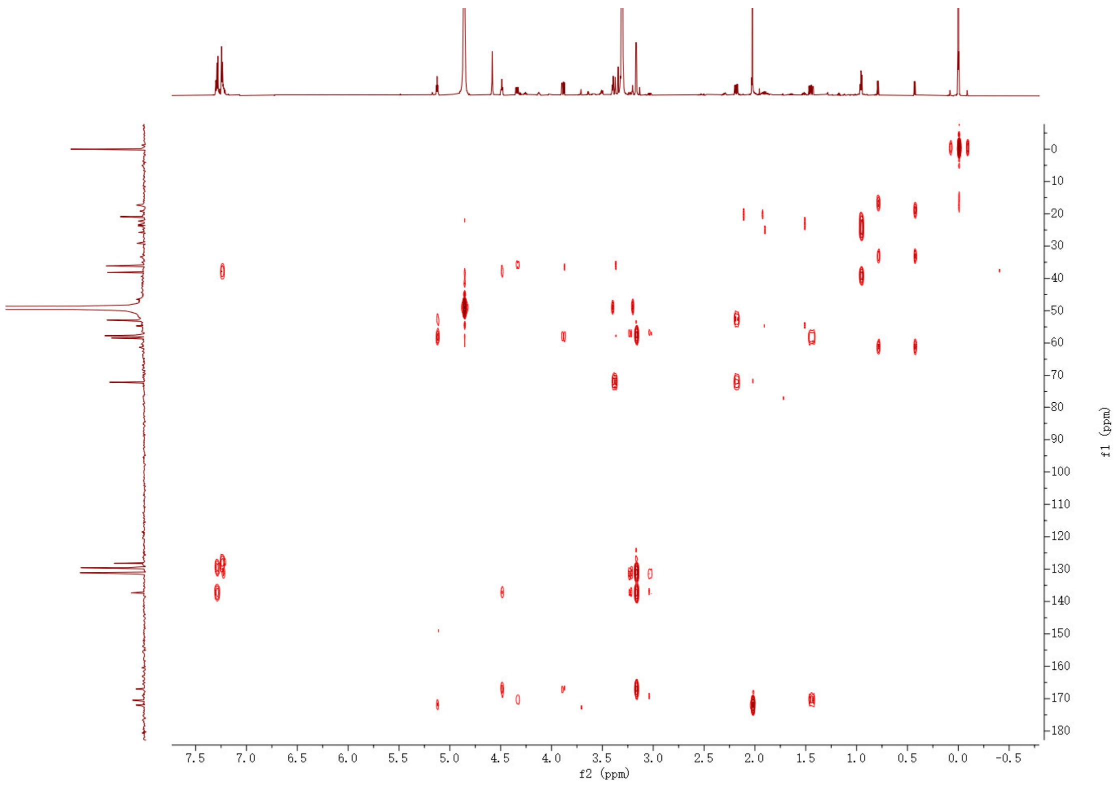

Figure S11. ^{1}H–^{1}H COSY spectrum of compound **1**

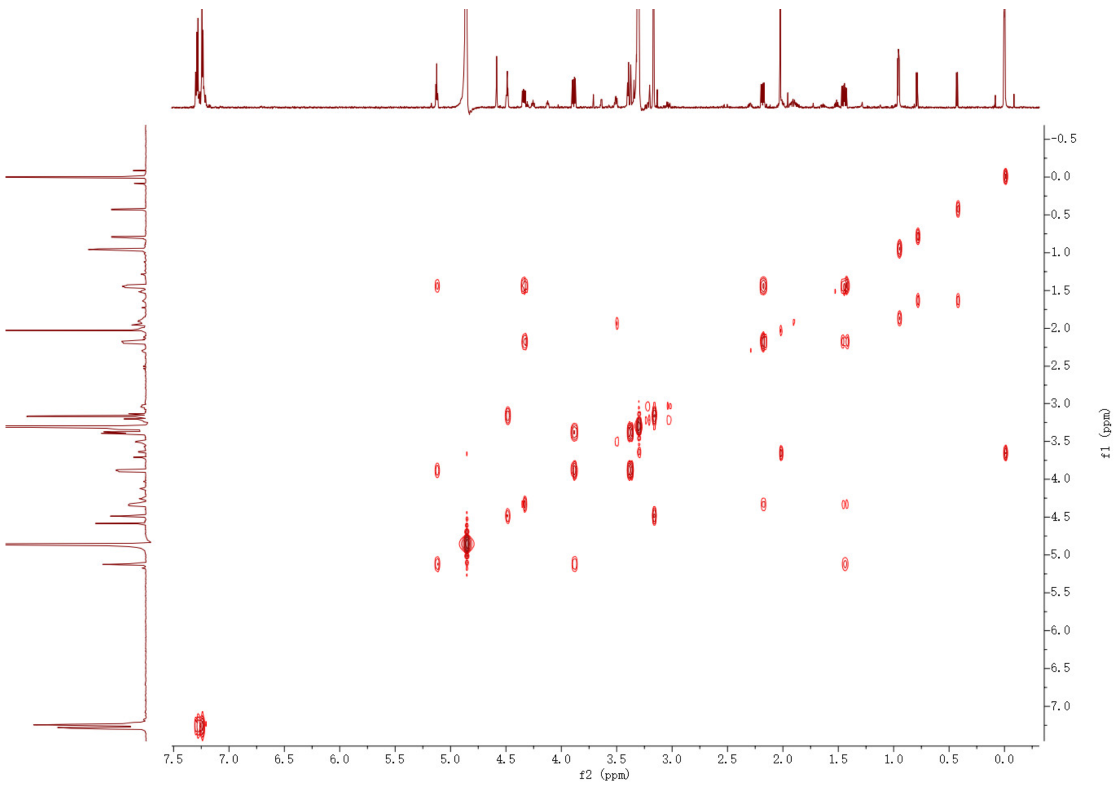

Figure S12. NOESY spectrum of compound **1**

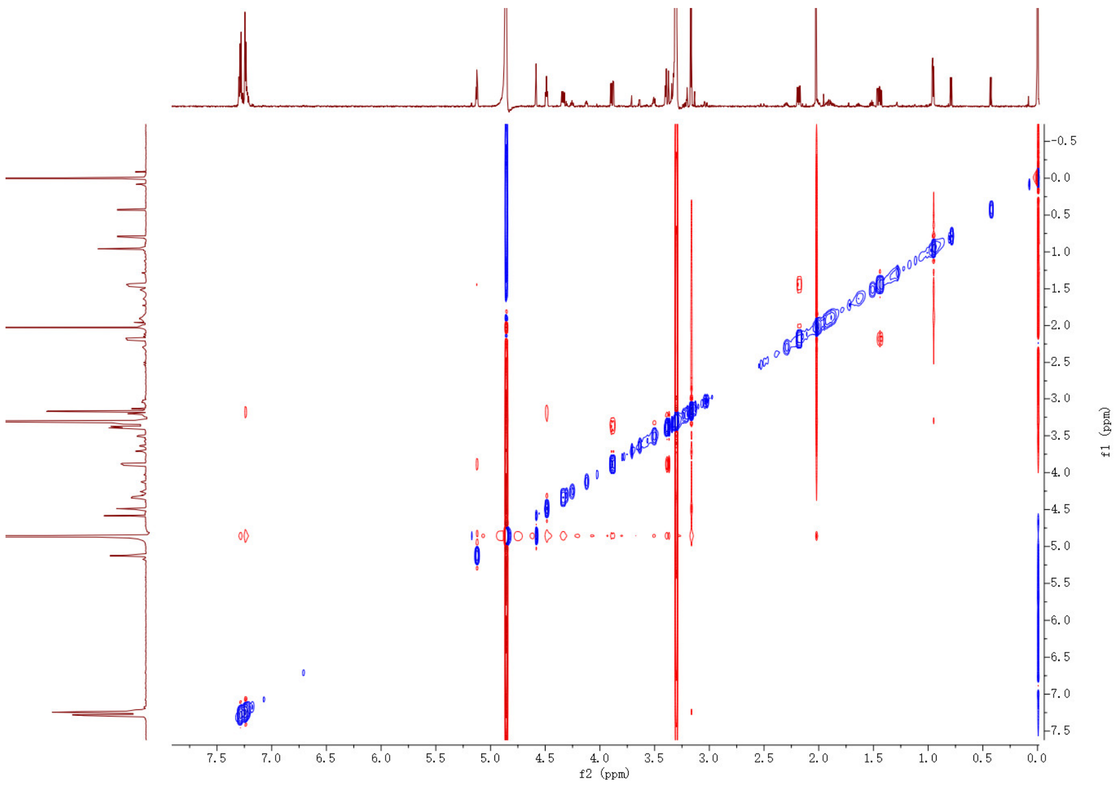

Figure S13. UV spectrum of compound **1**

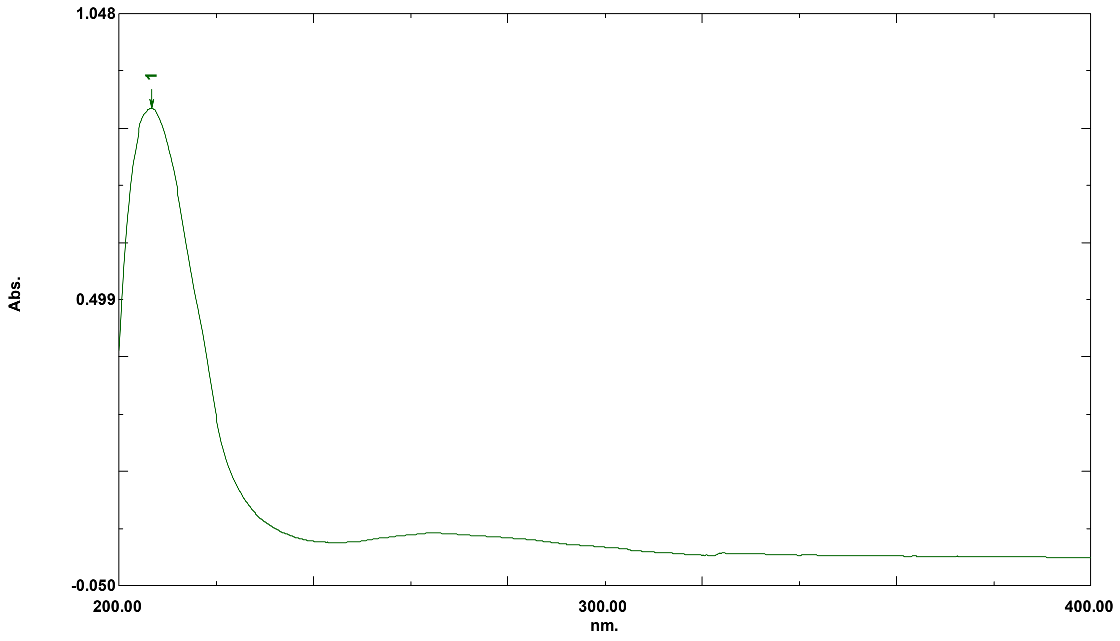

No.	wavelength (nm)	Abs
1	206.80	0.867

Figure S14. IR spectrum of compound **1**

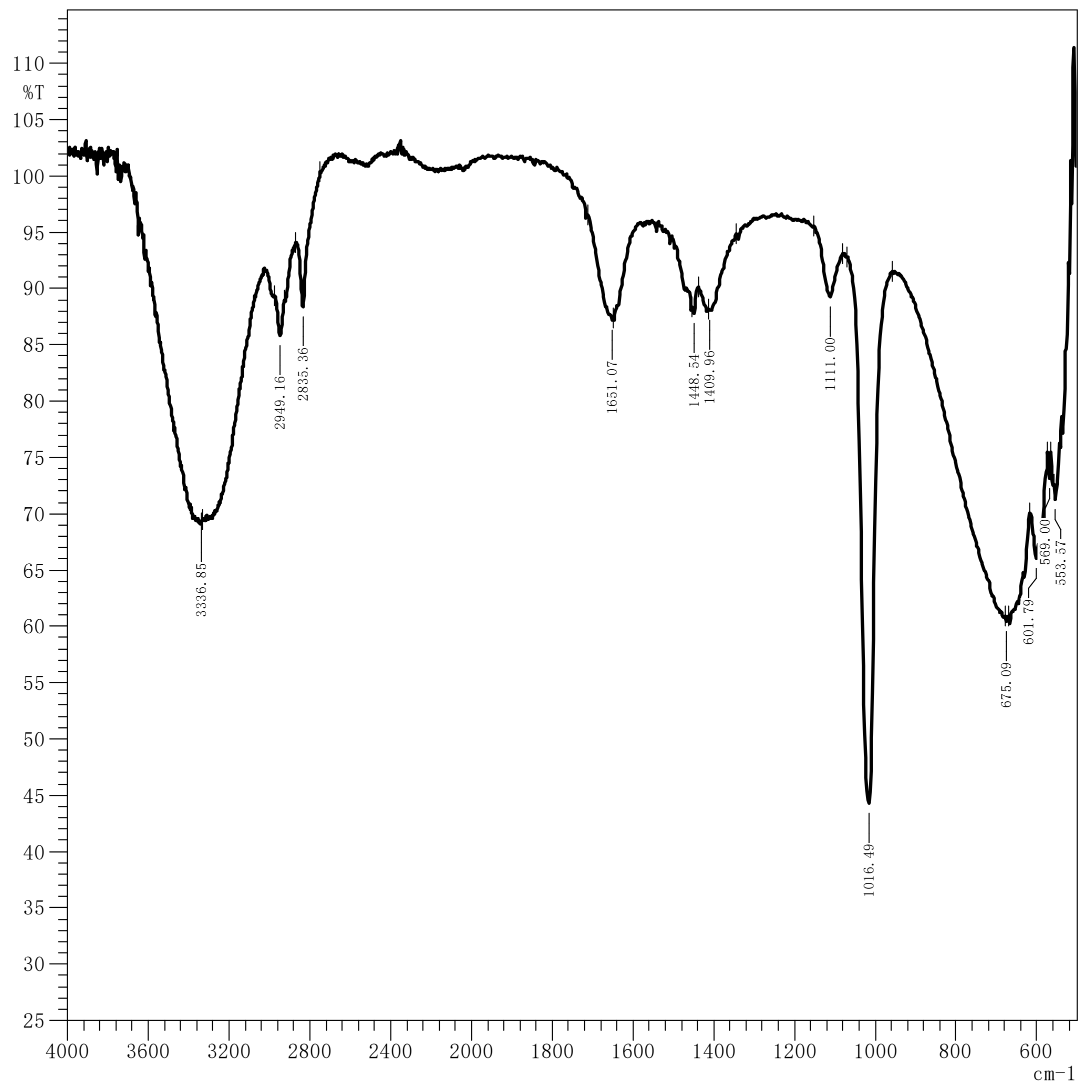

Table S5. ^{1}H and ^{13}C NMR data of compounds **2**–**3** (Methanol-d_4, δ in ppm, J in Hz).

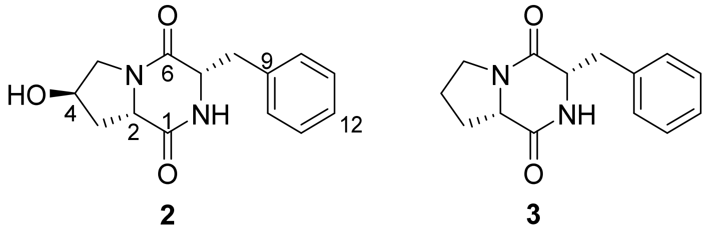

Position	**2**[a]		**3**[b]	
	δ_H	δ_C	δ_H	δ_C
1		171.3, s		170.9, s
2	4.36 (ddd, 11.8, 6.0, 2.0)	58.4, d	4.07 (ddd, 10.8, 6.4, 1.9)	60.1, d
3	2.06 (ddt, 12.9, 5.8, 1.3),<br>1.37 (ddd, 13.0, 11.7, 4.6)	38.0, t	2.09 (dtd, 12.3, 6.3, 4.4),<br>1.81 (overlapped)	29.4, t
4	4.28 (t, 4.8)	68.6, d	1.81 (overlapped), 1.23 (m)	22.8, t
5	3.70 (dd, 12.9, 5.2),<br>3.29 (d, 13.1)	55.3, t	3.54 (dt, 11.9, 8.3),<br>3.36 (m)	46.0, t
6		167.1, s		166.9, s
7	4.48 (td, 5.1, 1.9)	57.6, d	4.44 (td, 5.1, 1.9)	57.7, d
8	3.17 (m)	38.9, t	3.16 (m)	38.2, t
9		137.4, s		137.4, s
10, 14	7.21-7.29 (overlapped)	131.1, d	7.21-7.30 (overlapped)	131.1, d
11, 13	7.21-7.29 (overlapped)	129.5, d	7.21-7.30 (overlapped)	129.5, d
12	7.21-7.29 (overlapped)	128.1, d	7.21-7.30 (overlapped)	128.1, d

[a] ^{1}H and ^{13}C NMR data were recorded at 700 MHz and 176 MHz, respectively.

[b] ^{1}H and ^{13}C NMR data were recorded at 500 MHz and 126 MHz, respectively.

Table S6. ^{1}H and ^{13}C NMR data of compounds **4**–**5** (Methanol-d_4, δ in ppm, J in Hz).

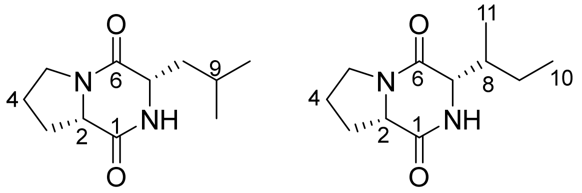

4 **5**

Position	**4**[a]		**5**[a]	
	δ_H	δ_C	δ_H	δ_C
1		172.8, s		167.6, s
2	4.30 (ddd, 9.3, 7.0, 1.8)	60.3, d	4.21 (m)	61.3, d
3	2.33 (m), 1.92-2.09 (overlapped)	29.1, t	2.06-2.46 (overlapped)	29.6, t
4	1.92-2.09 (overlapped)	23.7, t	2.06-2.46 (overlapped)	23.3, t
5	3.55 (m)	46.5, t	3.63-3.95 (overlapped)	46.2, t
6		168.9, s		172.5, s
7	4.16 (m)	54.6, d	4.32 (m)	60.0, d
8	1.92-2.09 (overlapped), 1.55 (m)	39.4, t	2.06-2.46 (overlapped)	37.1, d
9	1.92-2.09 (overlapped)	25.8, d	1.57 (m), 1.46 (m)	25.5, t
10	1.00 (d, 6.3)	23.4, q	1.06 (overlapped)	12.6, q
11	0.99 (d, 6.2)	22.3, q	1.20 (overlapped)	15.6, q

[a] ^{1}H and ^{13}C NMR data were recorded at 500 MHz and 126 MHz, respectively.

Table S7. ^{1}H and ^{13}C NMR data of compound **6** (Methanol-d_4, δ in ppm, J in Hz).

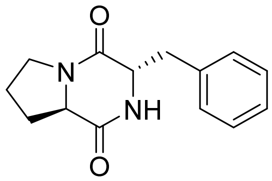

6

	**6**[a]	
Position	δ_H	δ_C
1		171.3, s
2	2.66 (10.7, 6.3)	59.1, d
3	2.07 (m), 1.70 (m)	29.8, t
4	1.93 (m), 1.64 (overlapped)	22.5, t
5	3.56 (dt, 12.2, 8.5), 3.35 (m)	46.2, t
6		167.4, s
7	4.23 (t, 4.7)	59.8, d
8	3.22 (dd, 13.7, 4.9), 3.03 (dd, 13.7, 4.7)	41.0, t
9		136.7, s
10, 14	7.22 (dd, 6.6, 3.0)	131.3, d
11, 13	7.33 (overlapped)	129.7, d
12	7.33 (overlapped)	128.5, d

[a] ^{1}H and ^{13}C NMR data were recorded at 500 MHz and 126 MHz, respectively.

Table S8. ^{1}H and ^{13}C NMR data of compounds **7–8** (Methanol-d_4, δ in ppm, J in Hz).

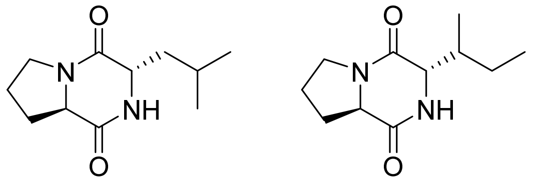

7 **8**

Position	**7**[a]		**8**[a]	
	δ_H	δ_C	δ_H	δ_C
1		171.6, s		171.6, s
2	4.30 (dd, 9.8, 6.6)	59.4, d	4.27 (dd, 9.9, 6.4)	59.8, d
3	2.37 (m), 2.06 (m)	29.9, t	2.38 (m), 2.05 (m)	30.3, t
4	1.96 (overlapped)	23.1, t	1.93 (overlapped)	22.9, t
5	3.61 (m), 3.53 (m)	46.7, t	3.63 (m), 3.52 (m)	46.8, t
6		169.1, s		167.9, s
7	3.88 (dd, 9.5, 5.4)	57.1, d	3.71 (d, 6.3)	63.5, d
8	1.71 (ddd, 13.5, 9.5, 5.5), 1.61 (ddd, 13.7, 8.5, 5.4)	43.7, t	1.93 (overlapped)	41.0, d
9	1.80 (m)	25.6, d	1.63 (m), 1.26 (m)	26.1, t
10	1.03 (d, 6.6)	23.4, q	0.98 (t, 7.4)	11.6, q
11	1.00 (d, 6.6)	22.0, q	1.03 (d, 6.9)	15.7, q

[a] ^{1}H and ^{13}C NMR data were recorded at 500 MHz and 126 MHz, respectively.

Table S9. ^{1}H and ^{13}C NMR data of compounds **9–10** (Methanol-*d*4, δ in ppm, J in Hz).

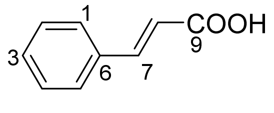

9

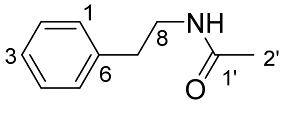

10

	**9**[a]			**10**[a]	
Position	δ_H	δ_C	Position	δ_H	δ_C
1, 5	7.59 (dd, 7.5, 2.1)	129.2, d	1, 5	7.27 (m)	129.5, d
2, 4	7.39 (overlapped)	130.0, d	2, 4	7.21 (m)	129.8, d
3	7.39 (overlapped)	131.3, d	3	7.18 (m)	127.4, d
6		136.0, s	6		140.6, s
7	7.65 (d, 16.0)	145.9, d	7	2.78 (t, 7.4)	36.5, t
8	6.48 (d, 16.0)	120.0, d	8	3.38 (m)	42.2, t
9		170.9, d	1'		173.3, s
			2'	1.90 (s)	22.5, q

[a] ^{1}H and ^{13}C NMR data were recorded at 700 MHz and 176 MHz, respectively.

Table S10. ^{1}H and ^{13}C NMR data of compound **11** (Chloroform-*d*, δ in ppm, J in Hz).

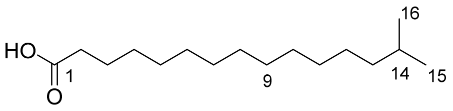

11

Position	**11**	
	δ_H	δ_C
1		180.5, s
2	2.38 (t, 7.5)	34.1, t
3	1.67 (m)	24.7, t
4-11	1.29-1.39 (overlapped)	29.1-30.0, t
12	1.29-1.39 (overlapped)	27.5, t
13	1.19 (m)	39.1, t
14	1.55 (m)	28.0, d
15	0.90 (d, 6.7)	22.7, q
16	0.90 (d, 6.7)	22.7, q

^{1}H and ^{13}C NMR data were recorded at 500 MHz and 126 MHz, respectively.

Table S11. ^{1}H and ^{13}C NMR data of compounds **12–13** (Chloroform-*d*, δ in ppm, *J* in Hz).

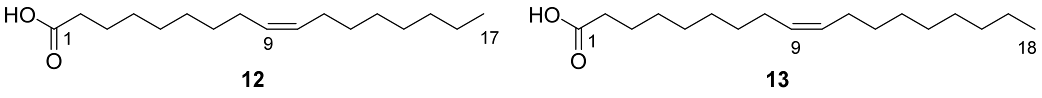

Position	**12**[a]		**13**[a]	
	δ_H	δ_C	δ_H	δ_C
1		180.5, s		180.4, s
2	2.38 (t, 7.5)	34.2, t	2.38 (t, 7.5)	34.2, t
3	1.67 (m)	24.7, t	1.67 (m)	24.7, t
4	1.29-1.38 (overlapped)	29.1-29.3, t	1.30-1.38 (overlapped)	29.0-29.1, t
5	1.29-1.38 (overlapped)	29.1-29.3, t	1.30-1.38 (overlapped)	29.0-29.1, t
6	1.29-1.38 (overlapped)	29.1-29.3, t	1.30-1.38 (overlapped)	29.4-29.6, t
7	1.29-1.38 (overlapped)	29.7-29.8, t	1.30-1.38 (overlapped)	29.7-29.8, t
8	2.05 (overlapped)	27.2-27.3, t	2.05 (overlapped)	27.2-27.3, t
9	5.38 (overlapped)	129.7, d	5.38 (overlapped)	129.8, d
10	5.38 (overlapped)	130.0, d	5.38 (overlapped)	130.0, d
11	2.05 (overlapped)	27.2-27.3, t	2.05 (overlapped)	27.2-27.3, t
12	1.29-1.38 (overlapped)	29.7-29.8, t	1.30-1.38 (overlapped)	29.7-29.8, t
13	1.29-1.38 (overlapped)	29.1-29.3, t	1.30-1.38 (overlapped)	29.4-29.6, t
14	1.29-1.38 (overlapped)	29.1-29.3, t	1.30-1.38 (overlapped)	29.4-29.6, t
15	1.29-1.38 (overlapped)	31.9, t	1.30-1.38 (overlapped)	29.0-29.1, t
16	1.29-1.38 (overlapped)	22.7, t	1.30-1.38 (overlapped)	32.0, t
17	0.91 (overlapped)	14.1, q	1.30-1.38 (overlapped)	22.7, t
18			0.92 (t, 6.8)	14.1, q

[a] ^{1}H and ^{13}C NMR data were recorded at 500 MHz and 126 MHz, respectively.

Table S12. ^{1}H and ^{13}C NMR data of compounds **14–15** (Chloroform-*d*, δ in ppm, J in Hz).

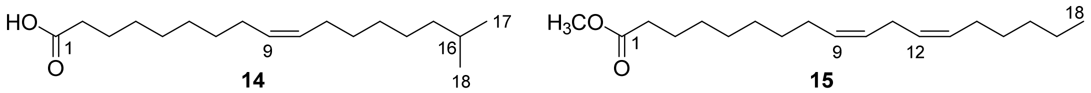

14 **15**

Position	**14**[a]		**15**[b]	
	δ_H	δ_C	δ_H	δ_H
1		179.8, s		174.3, s
2	2.39 (t, 7.5)	34.0, t	2.30 (t, 7.6)	34.1, t
3	1.67 (m)	24.7, t	1.62 (m)	25.0, t
4	1.29-1.39 (overlapped)	29.0-29.2, t	1.26-1.37 (overlapped)	29.1-29.6, t
5	1.29-1.39 (overlapped)	29.0-29.2, t	1.26-1.37 (overlapped)	29.1-29.6, t
6	1.29-1.39 (overlapped)	29.0-29.2, t	1.26-1.37 (overlapped)	29.1-29.6, t
7	1.29-1.39 (overlapped)	29.6-29.8, t	1.26-1.37 (overlapped)	29.1-29.6, t
8	2.05 (overlapped)	27.2-27.3, t	2.05 (overlapped)	27.2, t
9	5.38 (overlapped)	129.7, d	5.31-5.40 (overlapped)	130.0, d
10	5.38 (overlapped)	130.0, d	5.31-5.40 (overlapped)	127.9, d
11	2.05 (overlapped)	27.2-27.3, t	2.77 (t, 7.0)	25.6, t
12	1.29-1.39 (overlapped)	29.6-29.8, t	5.31-5.40 (overlapped)	128.0, d
13	1.29-1.39 (overlapped)	29. 6-29.8, t	5.31-5.40 (overlapped)	130.2, d
14	1.29-1.39 (overlapped)	27.2-27.3, t	2.05 (overlapped)	27.2, t
15	1.19 (m)	39.0, t	1.26-1.37 (overlapped)	29.1-29.6, t
16	1.55 (m)	28.0, d	1.26-1.37 (overlapped)	31.5, t
17	0.90 (d, 6.6)	22.7, q	1.26-1.37 (overlapped)	22.6, t
18	0.90 (d, 6.6)	22.7, q	0.89 (t, 7.0)	14.1, q
OCH_3			3.67 (s)	51.5, q

[a] ^{1}H and ^{13}C NMR data were recorded at 500 MHz and 126 MHz, respectively.

[b] ^{1}H and ^{13}C NMR data were recorded at 700 MHz and 176 MHz, respectively.

Table S13. Inhibitory effects of compounds **1–15**, and acarbose against *α*-glucosidase

Compounds [a]	Inhibition	Compounds [a]	Inhibition
**1**	4.16%	**9**	6.58%
**2**	-7.96%	**10**	3.23%
**3**	0.58%	**11**	-2.77%
**4**	-9.70%	**12**	-39.03%
**5**	1.27%	**13**	-22.17%
**6**	-9.23%	**14**	-15.47%
**7**	-7.27%	**15**	-7.51%
**8**	-13.16%	Acarbose	99.65%

[a] The inhibition of compounds **1**–**15** at the final concentration of 166.7 μg/mL, acarbose at the final concentration of 8.3 μg/mL.

Figure S15. GC-MS spectrum of compound **12**

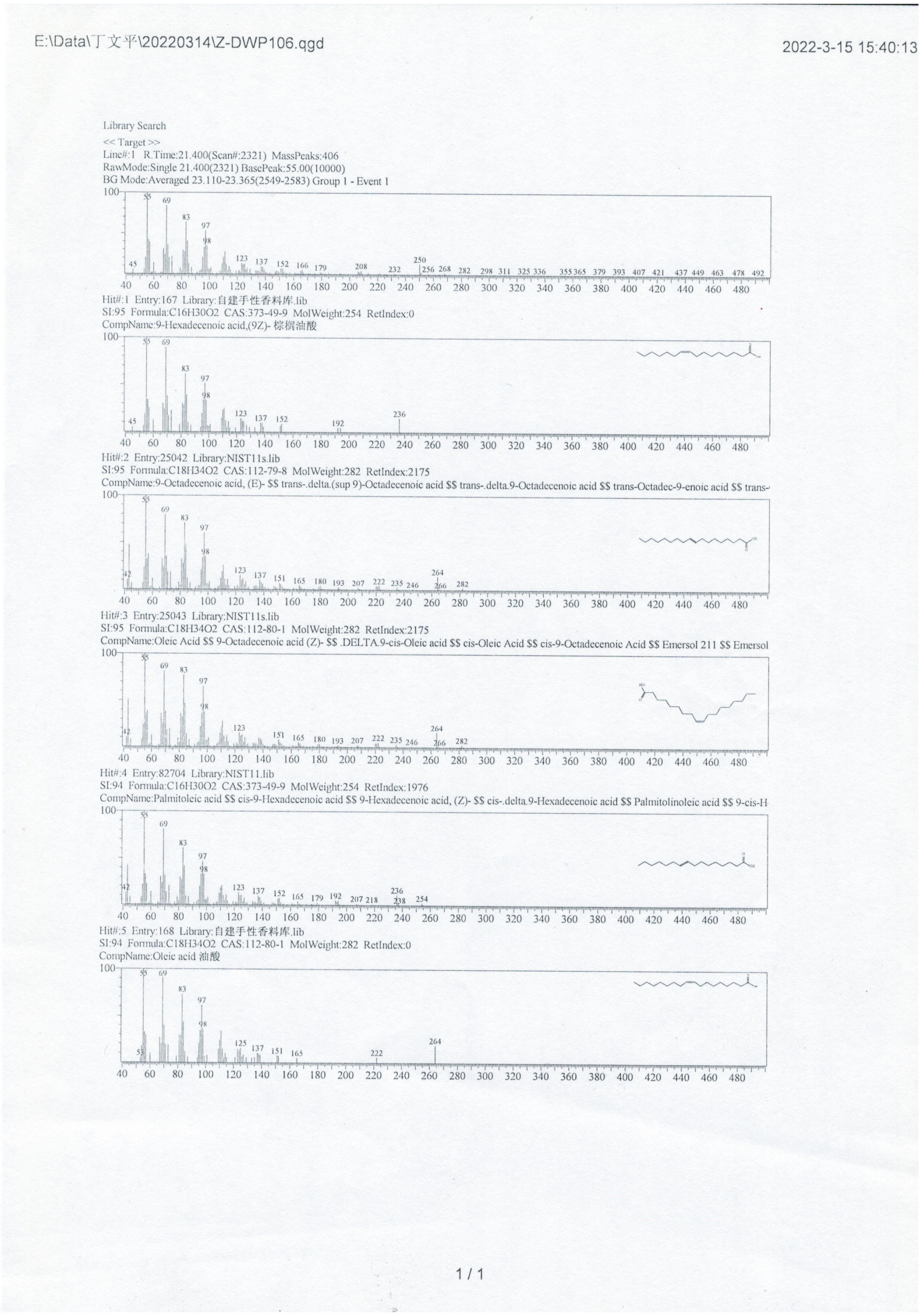

Figure S16. GC-MS spectrum of compound **13**

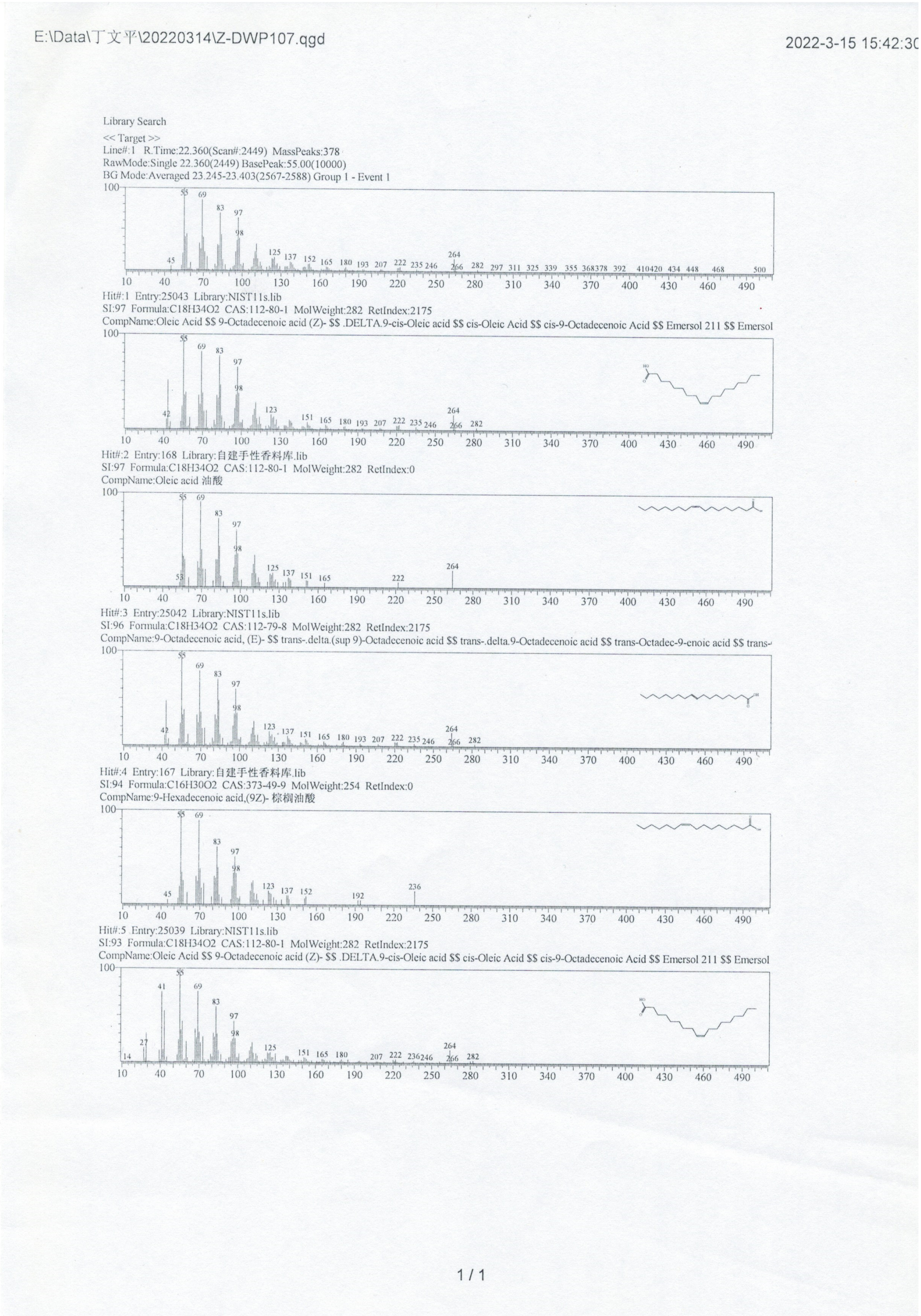

Figure S17. GC-MS spectrum of compound **14**

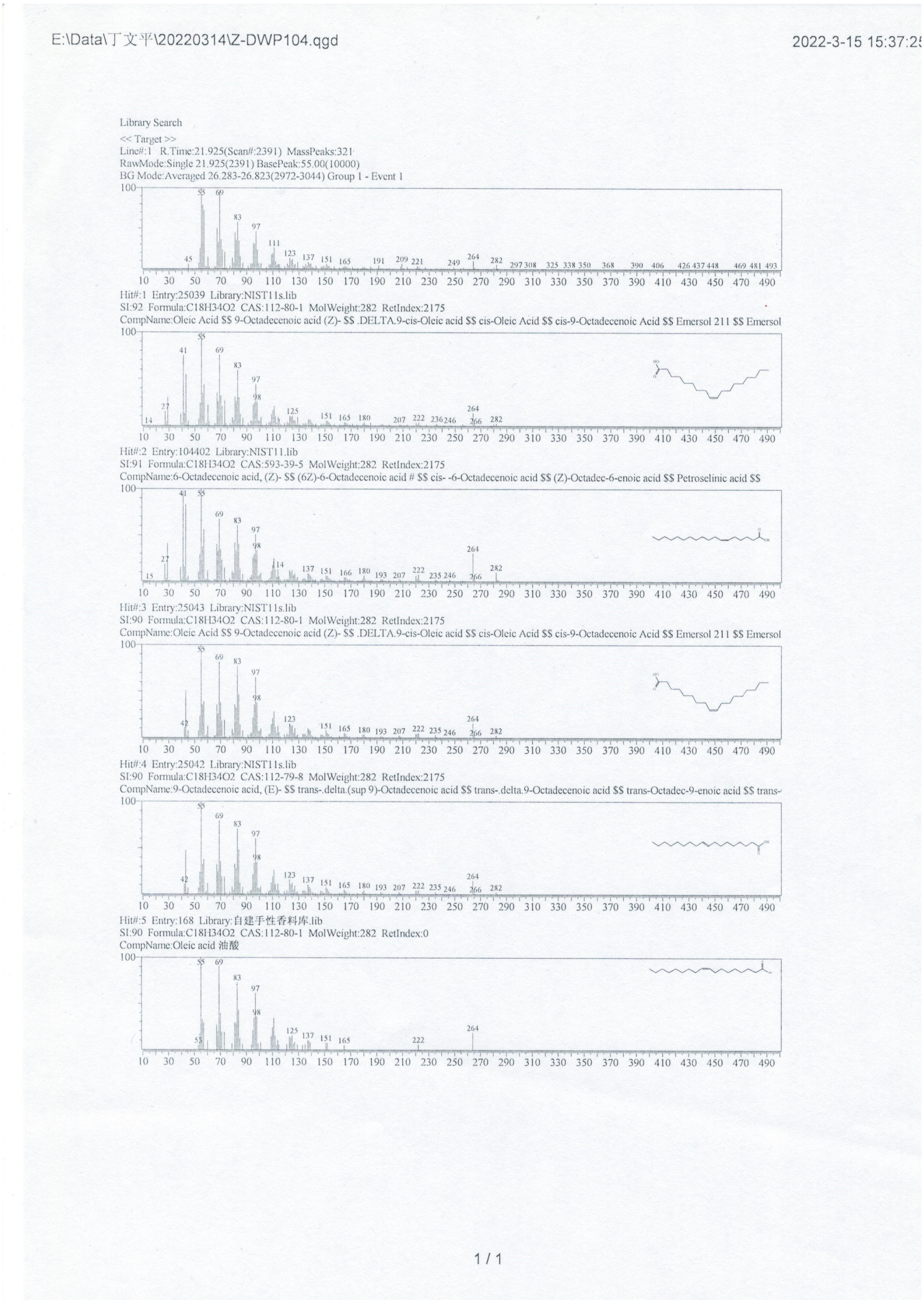

Figure S18. GC-MS spectrum of compound **15**

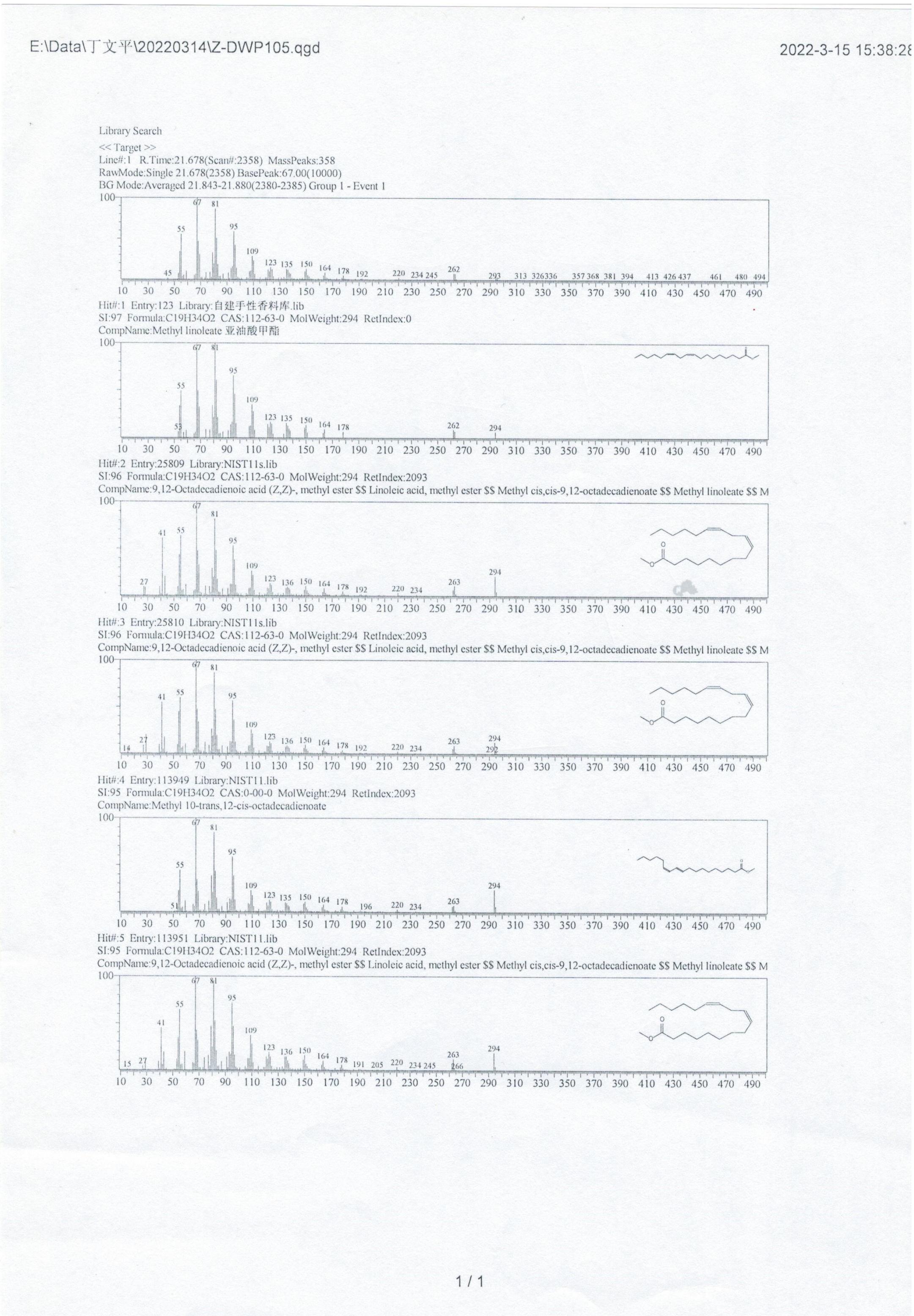

Figure S19. HPLC analysis profiles of Marfey's derivatives of **1**, **2**, and **5**–**7** at 340 nm

Marfey's derivatives of **1**

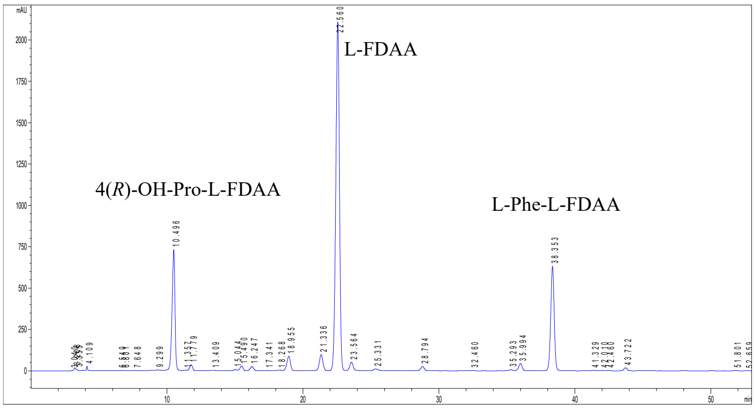

Marfey's derivatives of **2**

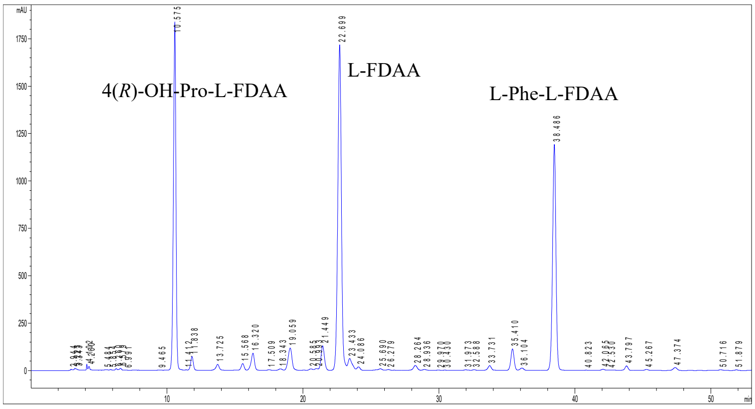

Marfey's derivatives of **5**

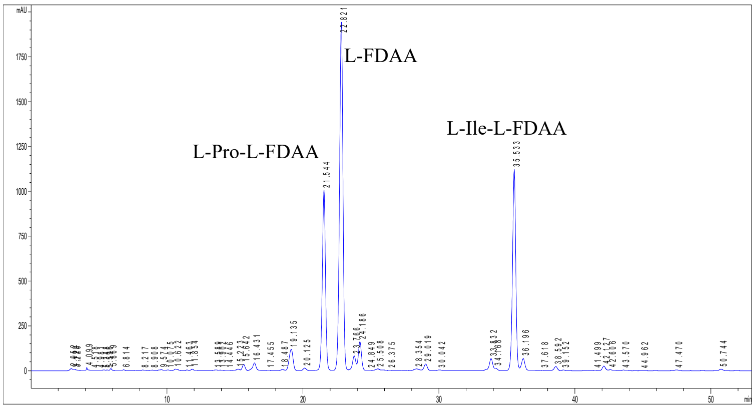

Marfey's derivatives of **6**

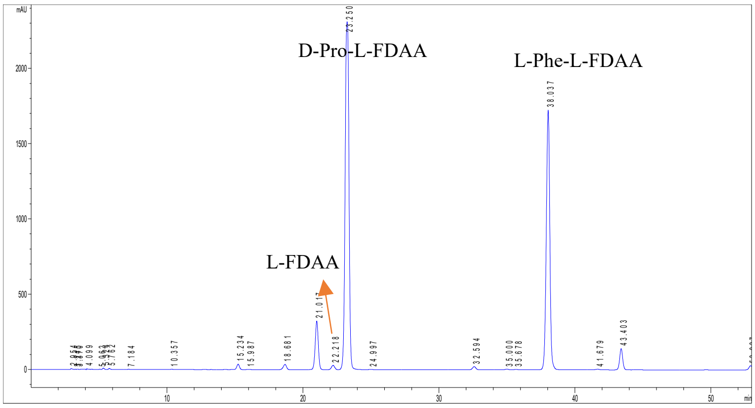

Marfey’s derivatives of **7**

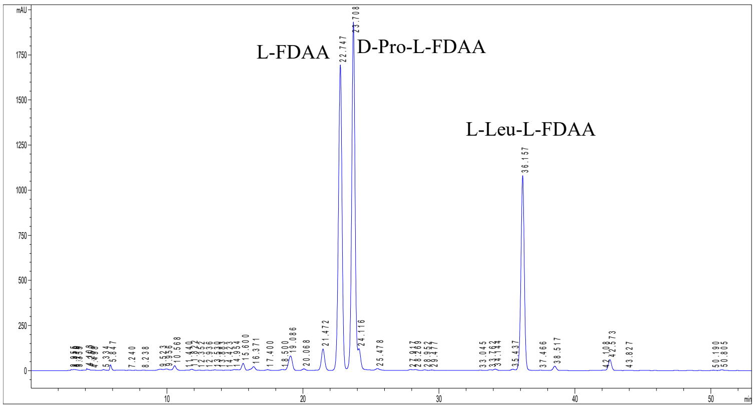

Supplement: Supplementary file 1 [file molecules-28-02133-s001.zip › molecules-2224139-supplementary.pdf]
